# AI-accelerated discovery of altermagnetic materials

**DOI:** 10.1093/nsr/nwaf066

**Published:** 2025-02-22

**Authors:** Ze-Feng Gao, Shuai Qu, Bocheng Zeng, Yang Liu, Ji-Rong Wen, Hao Sun, Peng-Jie Guo, Zhong-Yi Lu

**Affiliations:** Gaoling School of Artificial Intelligence, Renmin University of China, Beijing 100872, China; School of Physics, Renmin University of China, Beijing 100872, China; School of Physics, Renmin University of China, Beijing 100872, China; Gaoling School of Artificial Intelligence, Renmin University of China, Beijing 100872, China; School of Engineering Science, University of Chinese Academy of Sciences, Beijing 101408, China; Gaoling School of Artificial Intelligence, Renmin University of China, Beijing 100872, China; Gaoling School of Artificial Intelligence, Renmin University of China, Beijing 100872, China; School of Physics, Renmin University of China, Beijing 100872, China; School of Physics, Renmin University of China, Beijing 100872, China

**Keywords:** altermagnetism, pre-trained model, symmetry analysis, material discovery

## Abstract

Altermagnetism, a new magnetic phase, has been theoretically proposed and experimentally verified to be distinct from ferromagnetism and antiferromagnetism. Although altermagnets have been found to possess many exotic physical properties, the limited availability of known altermagnetic materials hinders the study of such properties. Hence, discovering more types of altermagnetic materials with different properties is crucial for a comprehensive understanding of altermagnetism and thus facilitating new applications in the next generation of information technologies, e.g. storage devices and high-sensitivity sensors. Since each altermagnetic material has a unique crystal structure, we propose an automated discovery approach empowered by an artificial intelligence (AI) search engine that employs a pre-trained graph neural network to learn the intrinsic features of the material crystal structure, followed by fine-tuning a classifier with limited positive samples to predict the altermagnetism probability of a given material candidate. Finally, we successfully discovered 50 new altermagnetic materials that cover metals, semiconductors and insulators, confirmed by first-principles electronic structure calculations. The wide range of electronic structural characteristics reveals that various novel physical properties manifest in these newly discovered altermagnetic materials, e.g. the anomalous Hall effect, anomalous Kerr effect and topological property. It is worth noting that we discovered four *i*-wave altermagnetic materials for the first time. Overall, the AI search engine performs much better than human experts and suggests a set of new altermagnetic materials with unique properties, outlining its potential for accelerated discovery of the materials with targeted properties.

## Teaser

This study introduces an Artificial Intelligence (AI) empowered search engine to accelerate the discovery of altermagnetic materials under the condition of limited labeled samples, demonstrating the potential of AI-driven methods to identify functional materials with novel properties.

## INTRODUCTION

Magnetic materials form a cornerstone of our modern information society. Generally, magnetism is categorized into ferromagnetism and antiferromagnetism. Recently, based on the spin group formalism [1], a new magnetic phase called altermagnetism has been theoretically proposed [2,3], which exhibits numerous novel physical properties [2–18], paving the path way for new avenues in the next generation of information technology. Both altermagnets and conventional antiferromagnets have compensated antiparallel spin sublattices, resulting in vanishing net magnetic momentum. The compensated antiparallel spin sublattices are connected by the spin symmetry $\lbrace C_2^{\perp }||I\rbrace$ or $\lbrace C_2^{\perp }||\tau \rbrace$ transformation for conventional antiferromagnets, but by the spin symmetry $\lbrace C_2^{\perp }||R_i\rbrace$ transformation for altermagnets [2]. Here, the symmetry operations at the left and right of the double vertical bar act only on the spin space and lattice space, respectively; the notation $C_2^{\perp }$ represents the $180^{\circ }$ rotation perpendicular to the spin direction; the notation $I, T, R_i$ and $\tau$ denote space inversion, time reversal, rotation/mirror and fractional translation operations, respectively. Because of the absence of spin symmetry $\lbrace C_2^{\perp }T||IT\rbrace$ or $\lbrace C_2^{\perp }||\tau \rbrace$, altermagnets have spin splitting in electronic bands. Unlike isotropic **k**-independent *s*-wave spin splitting in ferromagnets, altermagnets can form anisotropic **k**-dependent *d*-wave, *g*-wave and *i*-wave spin splitting according to different spin group symmetries [2]. Moreover, altermagnets not only have spin-splitting bands deriving from magnetic exchange interaction, which is the same as ferromagnets, but they also have unique extraordinary spin-splitting bands deriving from anisotropic electric crystal potential and magnetic exchange interaction [2]. In some altermagnets, the spin splitting can even have electronvolt magnitudes in parts of the Brillouin zone [2–4]. The anisotropic **k**-dependent spin splitting can result in a unique spin current by electrical means in *d*-wave altermagnets [5]. Based on the unique spin current, the spin-splitter torque in *d*-wave altermagnets was proposed in theory [5] and confirmed by experiments [6,7], which may circumvent limitations of spin-transfer torque (ferromagnets) and spin-orbit torque (conventional antiferromagnets or non-magnetic materials with strong spin-orbit coupling) in magnetic memory devices [5]. Meanwhile, the giant tunneling magnetoresistance [4] and giant piezomagnetism [19] can also be proposed in altermagnets based on the anisotropic **k**-dependent spin splitting. In the relativistic case, the time-reversal symmetry-breaking macroscopic phenomena, including quantum anomalous Hall [8], anomalous Hall [9,10] and anomalous Kerr effects [11], have been predicted by theories in altermagnets; moreover, the anomalous Hall effect has been supported by experiments [12,13].

On the other hand, magnetic topological phases and their exotic physical properties have recently attracted intensive experimental and theoretical attention. Very recently, some topological semimetal and insulator phases protected by spin group symmetry have been proposed in theory [20–26]. Considering the facts that altermagnets are described by spin group symmetry and that the symmetry landscape of spin space groups is more plentiful than that of the conventional magnetic space groups, more new magnetic topological phases and their exotic physical properties may thus be proposed theoretically in altermagnets. Nevertheless, altermagnets are hitherto in the early stage of research. Since there are many exotic physical properties that have been discovered and new physical phenomena to be discovered, altermagnets are bound to attract intensive theoretical and experimental attention in the near future. Very recently, based on spin group theory and known magnetic structures, 141 altermagnetic materials have been discovered [23]. However, known altermagnetic materials are still limited so far. Hence, there is an urgent need to discover more altermagnetic materials for a comprehensive understanding of altermagnetism, thus facilitating new applications in the next generation of information technology.

Conventional discovery methods primarily rely on the known magnetic structures and the corresponding spin space group. Such approaches are applicable only when the magnetic structure information is known a priori, which has a clear limitation if such information is missing. However, there exist over 90 000 magnetic materials documented in the Materials Project [27], among which only 2138 magnetic structures are known (see the MAGNDATA database [28]). The reason why the magnetic structures of only about 2% of magnetic materials have been determined is that it is indeed a non-trivial task that relies on extremely costly neutron scattering experimentation. Therefore, it is crucial to develop a method that breaks the bottleneck limitation of missing magnetic structure information, enabling the discovery of new altermagnetic materials without any prior knowledge of such information. On the other hand, the altermagnetic property is closely related to the material crystal structure, which provides a basis for the application of artificial intelligence (AI) methods to the discovery of altermagnetic materials. Moreover, the emerging AI technology has found many key applications in the discovery of materials [29]. For instance, AI was used for predicting organic compound synthesis in organic chemistry [30], planning chemical synthesis pathways [31], iterative synthesis of small molecules [32], accelerating the discovery of self-assembling peptides [33], designing eutectic solvents [34] and analyzing *de novo* protein mechanics and structures [35,36]. Recently, deep learning methods have been applied to the prediction of crystal materials with targeted properties [37,38]. These methods generally utilize a large amount of crystal structure data to train graph neural network (GNN) models in an end-to-end manner, without explicit reference to the physical laws underlying these material properties. The trained model could predict key physical properties of crystal materials, such as the formation energy and band gap, based on a rich training dataset containing over $10^4$ labeled samples [37]. However, such methods are not suitable for discovering altermagnetic materials, because of the fact that the known positive samples are limited.

**Figure 1.**
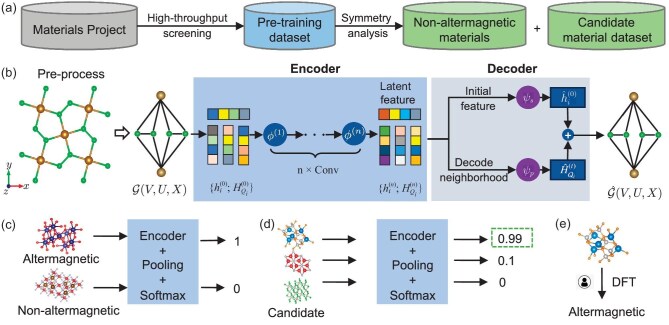
Workflow of the pre-trained model for searching altermagnetic materials. (a) Construction of candidate material datasets using high-throughput screening and symmetry analysis (see Fig. S1 for details). (b) The pre-training autoencoder framework for crystal materials. The input of the model is the crystal structure. Each crystal structure can be represented as a multiedge GNN. The encoder is built by the graph convolutional neural network. The decoder is built on the Waterstein neighborhood reconstruction. (c) The fine-tuning procedure with loading pre-training stage weight matrix. (d) The prediction procedure by inputting candidate materials. (e) Validation of the altermagnetic property via first-principles electronic structure calculations.

In this article, we introduce an AI search engine, as shown in Fig. 1, that combines deep model pre-training and fine-tuning techniques and physics-based approaches (e.g. symmetry analysis and first-principles electronic structure calculations) to discover new altermagnetic materials under the condition of limited labeled samples. In particular, we pre-train a self-supervised GNN [39] based on optimal transport theory [40] to learn the intrinsic features of the crystal structure of materials, and refine a downstream classifier with limited positive samples to predict the altermagnetism probability of a given material candidate. First, based on symmetry analysis, we constructed the pre-training dataset (containing 68 116 materials), fine-tuning the dataset (containing 25 739 materials, namely, 25 591 negative samples plus 148 positive samples) and candidate dataset (containing 42 377 materials) from the Materials Project [41]. Next, we pre-trained a GNN model (composed of an encoder and a decoder) for crystal materials, based on optimal transport theory. Once the pre-training was done, we fine-tuned the encoder on the fine-tuning dataset to obtain a classifier model. Then, the structured material information from the candidate dataset was input into the classifier model to quantify the probability as an indicator of whether each material is an altermagnetic material. We filtered out the materials with probabilities greater than 0.9 as the candidate altermagnetic materials. Finally, we employed the first-principles electronic structure calculations to estimate the ground magnetic structure of the candidate material to identify altermagnets. (It has been quite common to use first-principles electronic structure calculations, e.g. density functional theory (DFT), to predict the material property, which has been widely used in the community and proven to possess excellent alignment with experimental results for crystal materials [42–45].) Furthermore, the confirmed altermagnetic materials were added to the fine-tuning dataset for an iterative process of fine-tuning and classifier prediction, reinforcing the predictability of the model. The efficacy of this AI search engine has been well demonstrated.

Of the 91 649 total candidates, we discovered 50 new altermagnetic materials covering metals, semiconductors and insulators. The wide range of electronic structural properties implies that various novel physical properties appear in these newly discovered altermagnetic materials, e.g. the anomalous Hall effect, anomalous Kerr effect and topological property, as demonstrated in theoretical analyses. It is also worth noting that we discovered four *i*-wave altermagnetic materials for the first time, filling the gap in the literature. As a result, our proposed AI search engine successfully breaks the bottleneck limitation of existing discovery methods based on symmetry delimited rules, serves as a critical counterpart to such methods and is applicable to discovering new altermagnetic materials directly from a large set of candidates without any prior knowledge of the magnetic structure information. We conclude that the AI search engine suggests a set of new altermagnetic materials with unique properties, outlining its potential for accelerated discovery of the materials with targeting properties. We also discuss the pathway of developing pre-trained graph models for the discovery of other types of materials.

## RESULTS

### Dataset screening via symmetry analysis

Our goal is to screen altermagnetic materials from the Materials Project [27], which contains 154 718 crystal materials. Since this materials database includes both magnetic and non-magnetic materials, we first filtered out materials containing magnetic atoms. In this work, we considered materials with 3d transition metals or 4f rare earth elements. After filtering and de-duplication, we obtained 91 649 potential magnetic materials. Because of the complexity of the magnetic properties of materials with multiple magnetic atoms, we further excluded such materials, resulting in 68 116 potential magnetic materials, which constitute the pre-training dataset.

Altermagnetism is characterized by compensated antiparallel spin sublattices connected by the spin symmetry $\lbrace C_2^{\perp }||R_i\rbrace$ transformation, but not connected by the spin symmetry $\lbrace C_2^{\perp }||I\rbrace$ or $\lbrace C_2^{\perp }||\tau \rbrace$ transformation. Since the space groups $P1 (1)$ and $P\bar{1}(2)$ do not have $R_i$ symmetry, all materials with space groups $P1$ and $P\bar{1}$ symmetry are excluded from the pre-training dataset. If collinear antiferromagnets have type-IV magnetic space group symmetry, their compensated antiparallel spin sublattices must be connected by the spin symmetry $\lbrace C_2^{\perp }||\tau \rbrace$ transformation in a non-relativistic case. So all collinear antiferromagnetic materials with type-IV magnetic space group symmetry are conventional antiferromagnets but not altermagnets. Different from antiferromagnetic materials with the type-IV magnetic space group symmetry, the magnetic cell and crystal cell of materials with type-III magnetic space group symmetry are usually the same, which leads to these materials without the spin symmetry $\lbrace C_2^{\perp }||\tau \rbrace$. If the magnetic cell of a collinear antiferromagnet is a supercell, whereas its spin arrangement breaks the spin symmetry $\lbrace C_2^{\perp }||\tau \rbrace$, then the collinear antiferromagnet may be a supercell altermagnet [46]. Although there exist four known supercell altermagnetic materials, we do not consider this situation and exclude them in the positive samples. Since compensated antiparallel spin sublattices in altermagnets require the candidate magnetic materials to have an even number of magnetic atoms in their crystal primitive cell, we first ruled out 18 546 magnetic materials with an odd number of magnetic atoms in the primitive crystal cell from the pre-training dataset.

Furthermore, magnetic materials with type-III magnetic space group symmetry can be divided into two classes according to space-inversion symmetry. If the crystal structure of a collinear antiferromagnet has no combination of space-inversion and time-reversal symmetry, such a material must be altermagnetic. Otherwise, if the collinear antiferromagnetic material has only a pair of spin antiparallel magnetic atoms in the primitive crystal cell that are not located at invariant space-inversion points, the pair of spin antiparallel magnetic atoms must be connected by the spin symmetry $\lbrace C_2^{\perp }||I\rbrace$. This class collinear antiferromagnets are not altermagnetic materials (7045 in total). Therefore, based on symmetry analysis, we screened out 25 591 non-altermagnetic materials. These materials, along with the known 148 altermagnetic materials (e.g. as positive samples), constitute the fine-tuning dataset. By removing the 25 591 non-altermagnetic materials and positive samples from the pre-training dataset, we obtained the candidate dataset (42 377 materials). The aforementioned screening process is depicted in Fig. S1. In the following, we train a neural network to screen and predict altermagnetic materials from the candidate dataset.

### Pre-training the GNN for material discovery

Although AI methods have shown great potential for material screening and discovery, there still remain numerous challenges in the field of discovering altermagnetic materials that have not yet been accommodated in existing research practices. In particular, training a reliable predictive model under the condition of limited labels is intractable, e.g. the number of known altermagnetic materials, as positive samples (training labels), is limited (only 148 altermagnetic materials [3,23]). We address this challenge by introducing a pre-training and fine-tuning technique, which was first proposed in the natural language processing field [47], and subsequently demonstrated with remarkable capabilities for computer vision [48] and bioinformatics [49]. Such a technique pre-trains a self-supervised model first, then refines it for a specific downstream task with limited data, meanwhile maintaining a boosted performance. Since each crystal material has a unique structure-property relationship, e.g. the magnetic-property-like spin pattern is closely related to the crystal structure information, we hypothesize that there is a functional correspondence between the spin pattern and the crystal structure for a given material candidate. Hence, we represent the material crystal structures by multiedge graphs and establish a pre-trained neural network model to extract their corresponding latent features. The discovery of an altermagnetic material process is then treated as a downstream task by refining a classifier model based on limited positive samples (e.g. 148 available altermagnetic materials).

**Figure 2.**
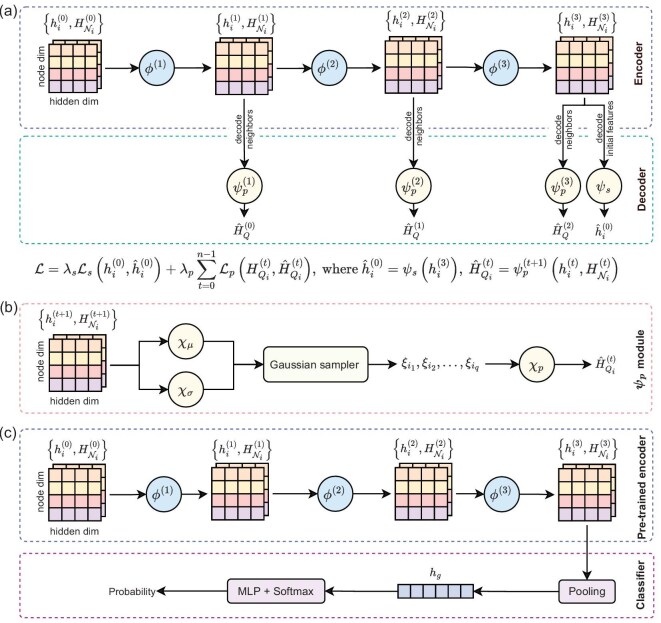
Network architectures of the auto-encoder and classifier. (a) Details of the auto-encoder model. The encoder consists of three graph convolution layers denoted by $\phi ^{(1)}, \phi ^{(2)}, \phi ^{(3)}$, whose inputs are node features $h_i^{(t)}$ and neighbor features $H_{\mathcal {N}_i}^{(t)}$, where $t=0,1,2$, respectively. The decoder is composed of a decoder module $\psi _s$ for reconstructing initial node features and three decoder modules $\psi _p^{(1)}, \psi _p^{(2)}, \psi _p^{(3)}$ for reconstructing a neighborhood set of node features. We minimize the weighted sum of the reconstruction loss functions for both decoder modules. (b) Details of the $\psi _p$ module in the decoder. The $\psi _p$ module includes three MLPs ($\chi _{\mu }, \chi _{\sigma }$ and $\chi _{p}$) and a Gaussian sampler, while the $\psi _s$ module is composed of a single MLP. (c) Details of the classifier model. The node features $h_i^{(0)}$ and the neighborhood set of node features $H_{\mathcal {N}_i}^{(0)}$ are fed into the pre-trained encoder. The output node features ${h}_i^{(3)}$ are then transformed to a latent vector ${h}_g$ by a pooling layer. Finally, another MLP and softmax module is designed to output the probability that quantifies whether the material is altermagnetic.

As detailed in the symmetry analysis above, we first construct the pre-training dataset and candidate material dataset based on high-throughput screening and symmetry analysis (see Fig. 1(a)). The pre-trained model is based on a GNN that leverages material crystal structure information [37], consisting of a graph convolutional network encoder, and a decoder that reconstructs graph features based on optimal transport theory [50]. Figure 1(b) depicts the schematic of the network, with the detailed architecture shown in Fig. 2. The process of inputting crystal structures into the model begins with a pre-processing stage, where the crystal structure information is transformed into a graph representation. Then, we pre-train the model based on the pre-training dataset that contains 68 116 materials, and then fine-tune the pre-trained model based on the fine-tuning dataset (148 altermagnetic materials plus 25 591 non-altermagnetic materials; see Fig. 1(c)). (Note that there are some biases in the negative sampling, but its influence on the predictive performance of our AI model is negligible. This is because the number of negative samples is significantly larger, being 172 times greater than the number of positive samples.) During the fine-tuning, we utilize the pre-trained encoder and employ up-sampling techniques (duplication and rotation) to balance the number of positive and negative samples for a binary classification task. Afterward, we can obtain the classifier model, which is then used to screen the altermagnetic materials (Fig. 1(d)). All possible candidate crystal structures (42 377) are input into the classifier model for prediction. The model provides a probability estimate for each sample, and we selected the material with a probability greater than 0.9 as the candidate material. Next, we utilize the first-principles electronic structure calculations (Fig. 1(e)) to verify whether the candidates are altermagnetic materials. Furthermore, once the new altermagnetic materials are verified and confirmed, we add the new one to the fine-tuning dataset and then re-perform the fine-tuning and prediction iteratively. Through four rounds of iteration and leveraging information from 148 known altermagnetic materials, we identified 50 new altermagnetic materials. A discussion of the model convergence and the spin patterns’ distinguish ability is provided in Note C within the online supplementary material. Additional information for the pre-trained model is given in Note A within the online supplementary material.

To demonstrate the capability of our pre-trained crystal material model, we fed all the candidate materials in batches into the pre-trained encoder that provides a corresponding latent space vector for each material. We utilized principal component analysis for dimensionality reduction and performed feature visualization and t-SNE visualization on the latent space vectors (see Fig. S2). The results show that the data in the candidate set have a clear clustering phenomenon after pre-training, which indicates that the pre-training process can group materials containing similar information together.

### Discovered altermagnetic materials

Based on the proposed AI search engine, we successfully discovered 50 new altermagnetic materials, including 16 metals and 34 insulators (see Table 1). The computational results for most of the newly discovered altermagnetic materials (e.g. the first 23 materials listed in Table 1) are shown in Note B and Figs S6–S17. Moreover, the *d*-wave, *g*-wave and *i*-wave altermagnets can be found in the predicted 50 altermagnetic materials shown in Table 1. In particular, we predicted four *i*-wave altermagnetic materials for the first time.

**Table 1.** Fifty newly discovered altermagnetic materials verified by electronic structure calculations.

					Materials	
Number	Material	Space group	Anisotropy	Conduction	Project ID	Reference
1	$\text{Nb}_2\text{Fe}\text{B}_2$	$P4$ /$mbm\hphantom{0}(127)$	*g*-wave	M	mp-1086660	[52]
2	$\text{Ta}_2\text{Fe}\text{B}_2$	$P4$ /$mbm\hphantom{0}(127)$	*g*-wave	M	mp-1095076	[53]
3	$\text{Nd}\text{B}_2\text{C}_2$	$P4$ /$mbm\hphantom{0}(127)$	*g*-wave	M	mp-5765	[51]
4	$\text{Mg}_2\text{Fe}\text{Ir}_5\text{B}_2$	$P4$ /$mbm\hphantom{0}(127)$	*g*-wave	M	mp-1188243	[54]
5	$\text{Mg}_2\text{Mn}\text{Ir}_5\text{B}_2$	$P4$ /$mbm\hphantom{0}(127)$	*g*-wave	M	mp-1189623	[54]
6	$\text{Mg}_2\text{Ni}\text{Ir}_5\text{B}_2$	$P4$ /$mbm\hphantom{0}(127)$	*g*-wave	M	mp-1188248	[54]
7	$\text{Sc}_2\text{V}\text{Ir}_5\text{B}_2$	$P4$ /$mbm\hphantom{0}(127)$	*g*-wave	M	mp-20524	[55]
8	$\text{Sc}_2\text{Mn}\text{Ir}_5\text{B}_2$	$P4$ /$mbm\hphantom{0}(127)$	*g*-wave	M	mp-1208987	[55]
9	$\text{Ca}\text{La}\text{Fe}\text{Ag}\text{O}_6$	$Pc\hphantom{0}(7)$	*d*-wave	M	mp-1641528	NA
10	$\text{Ca}\text{La}\text{Cr}_2\text{O}_6$	$Pmn2_1\hphantom{0}(31)$	*d*-wave	M	mp-1642123	NA
11	$\text{Ni}\text{F}_3$	$R\bar{3} c\hphantom{0}(167)$	*i*-wave	M	mp-561428	[56]
12	$\text{Gd}\text{B}_2\text{C}_2$	$P4/mbm\hphantom{0}(127)$	*g*-wave	M	mp-1080176	[57]
13	$\text{Ho}\text{B}_2\text{C}_2$	$P4/mbm\hphantom{0}(127)$	*g*-wave	M	mp-20410	[58]
14	$\text{Lu}\text{Cr}\text{O}_3$	$Pnma\hphantom{0}(62)$	*d*-wave	M	mp-755471	[59]
15	$\text{Ta}\text{Co}\text{B}_2$	$Pnma\hphantom{0}(62)$	*d*-wave	M	mp-1189690	NA
16	$\text{Nd}\text{Ru}\text{O}_3$	$Pnma\hphantom{0}(62)$	*d*-wave	M	mp-1200843	[60]
17	$\text{Fe}\text{H}\text{O}_2$	$Pmn2_1\hphantom{0}(31)$	*d*-wave	I	mp-510670	[61]
18	$\text{Na}\text{Fe}\text{O}_2$	$Pna2_1\hphantom{0}(33)$	*d*-wave	I	mp-21060	[62]
19	$\text{Na}\text{Fe}\text{O}_2$	$P4_12_12\hphantom{0}(92)$	*d*-wave	I	mp-21880	[63]
20	$\text{Mn}\text{O}_2$	$Pnma\hphantom{0}(62)$	*d*-wave	I	mp-19326	[64]
21	$\text{Mn}\text{O}_2$	$I4/m\hphantom{0}(87)$	*d*-wave	I	mp-19395	[65]
22	$\text{Ca}_3\text{Cr}_2\text{O}_7$	$Cmc2_1\hphantom{0}(36)$	*d*-wave	I	mp-1575873	NA
23	$\text{Zr}\text{Cr}\text{O}_3$	$Pnma\hphantom{0}(62)$	*d*-wave	I	mp-755055	NA
24	$\text{Zr}\text{Mn}\text{O}_3$	$R3c\hphantom{0}(161)$	*i*-wave	I	mp-754513	NA
25	$\text{V}\text{F}_3$	$R\bar{3} c\hphantom{0}(167)$	*i*-wave	I	mp-559931	[56]
26	$\text{Cr}\text{F}_3$	$R\bar{3} c\hphantom{0}(167)$	*i*-wave	I	mp-560338	[56]
27	$\text{Mn}\text{O}$	$P6_3mc\hphantom{0}(186)$	*g*-wave	I	mp-999539	[66]
28	$\text{Ca}\text{Mn}\text{N}_2$	$P6_3/mmc\hphantom{0}(194)$	*g*-wave	I	mp-1246377	NA
29	$\text{Ba}_2\text{Fe}\text{Ge}_2\text{O}_7$	$P\overline{4}2_1m\hphantom{0}(113)$	*g*-wave	I	mp-1190820	[67]
30	$\text{Ba}_2\text{Co}\text{Si}_2\text{O}_7$	$P\overline{4}2_1m\hphantom{0}(113)$	*g*-wave	I	mp-510015	[68]
31	$\text{Sr}_2\text{Co}\text{Ge}_2\text{O}_{7}$	$P\overline{4}2_1m\hphantom{0}(113)$	*g*-wave	I	mp-1191317	[69]
32	$\text{V}\text{F}_4$	$P2_1/c\hphantom{0}(14)$	*d*-wave	I	mp-760030	NA
33	$\text{Ca}_2\text{Co}\text{Te}\text{O}_{6}$	$P2_1/c\hphantom{0}(14)$	*d*-wave	I	mp-552051	[70]
34	$\text{Ni}\text{F}_2$	$Pnnm\hphantom{0}(58)$	*d*-wave	I	mp-556324	[71]
35	$\text{Li}\text{Fe}_2\text{F}_{6}$	$P4_2nm\hphantom{0}(102)$	*d*-wave	I	mp-557403	[72]
36	$\text{Fe}\text{H}\text{O}_2$	$P2_12_12_1\hphantom{0}(19)$	*d*-wave	I	mp-625251	NA
37	$\text{Ca}\text{Mn}\text{O}_3$	$Pnma\hphantom{0}(62)$	*d*-wave	I	mp-19201	[73]
38	$\text{Ca}\text{V}\text{O}_3$	$Pnma\hphantom{0}(62)$	*d*-wave	I	mp-22608	[74]
39	$\text{La}\text{Fe}\text{O}_3$	$Pnma\hphantom{0}(62)$	*d*-wave	I	mp-22590	[75]
40	$\text{La}\text{V}\text{O}_3$	$Pnma\hphantom{0}(62)$	*d*-wave	I	mp-19350	[76]
41	$\text{Mn}\text{Se}\text{O}_4$	$Pnma\hphantom{0}(62)$	*d*-wave	I	mp-817982	[77]
42	$\text{Na}\text{Pr}_2\text{Os}\text{O}_6$	$P2_1/c\hphantom{0}(14)$	*d*-wave	I	mp-20009	[78]
43	$\text{Na}\text{Pr}_2\text{Ru}\text{O}_6$	$P2_1/c\hphantom{0}(14)$	*d*-wave	I	mp-542512	[79]
44	$\text{Nd}\text{Rh}\text{O}_3$	$Pnma\hphantom{0}(62)$	*d*-wave	I	mp-4582	[80]
45	$\text{Pr}\text{Ru}\text{O}_3$	$Pnma\hphantom{0}(62)$	*d*-wave	I	mp-20186	[81]
46	$\text{Sc}\text{V}\text{O}_3$	$Pnma\hphantom{0}(62)$	*d*-wave	I	mp-756546	[82]
47	$\text{Sm}\text{Rh}\text{O}_3$	$Pnma\hphantom{0}(62)$	*d*-wave	I	mp-3317	[83]
48	$\text{Ca}\text{La}\text{Cr}\text{Mo}\text{O}_6$	$Pc\hphantom{0}(7)$	*d*-wave	I	mp-1640189	NA
49	$\text{La}_2\text{Mn}\text{Rh}\text{O}_6$	$P2_1/c\hphantom{0}(14)$	*d*-wave	I	mp-1223338	NA
50	$\text{Li}\text{Fe}\text{F}_4$	$P2_1/c\hphantom{0}(14)$	*d*-wave	I	mp-755632	NA

The table also lists the non-magnetic space group, even-parity wave anisotropy and metal(M) and insulator(I) conduction type. Altermagnetic $\text{Nd}\text{B}_2\text{C}_2$ is confirmed by previous neutron scattering experiments [51] and our symmetry analysis. Here ‘NA’ indicates that this material has not been experimentally synthesized. The information of whether a material is a metal or an insulator is confirmed by DFT calculations.

The 16 metallic altermagnetic materials can be divided into two classes according to whether the integral of the Berry curvature of the occupied states over the Brillouin zone is zero, which depends on the symmetry of the altermagnetic materials. Since the easy magnetization axes of these materials are in the *x*-*y* plane, the eight metallic altermagnetic materials $\text{Nb}_2\text{Fe}\text{B}_2$, $\text{Ta}_2\text{Fe}\text{B}_2$, $\text{Nd}\text{B}_2\text{C}_2$, $\text{Mg}_2\text{Fe}\text{Ir}_5\text{B}_2$, $\text{Mg}_2\text{Mn}\text{Ir}_5\text{B}_2$, $\text{Mg}_2\text{Ni}\text{Ir}_5\text{B}_2$, $\text{Sc}_2\text{V}\text{Ir}_5\text{B}_2$, $\text{Sc}_2\text{Mn}\text{Ir}_5\text{B}_2$ have non-zero Berry curvature for the integral of the occupied states over the Brillouin zone according to magnetic point group symmetry, implying that odd-under-time-reversal responses (e.g. anomalous Hall and Kerr effects) can be realized in these materials. In particular, the calculated intrinsic anomalous Hall conductance of altermagnet $\text{Nb}_2\text{Fe}\text{B}_2$ is $-100\, \Omega ^{-1} {\rm cm}^{-1}$ [84], which is the same order of magnitude as those of ferromagnetic metals. Since the three altermagnetic materials $\text{Nd}\text{B}_2\text{C}_2$, $\text{Sc}_2\text{Mn}\text{Ir}_5\text{B}_2$, $\text{Mg}_2\text{Ni}\text{Ir}_5\text{B}_2$, whose easy magnetization axes are in the *z* direction, have zero Berry curvature for the integral of the occupied states over the Brillouin zone, the anomalous Hall effect is not observed.

**Figure 3.**
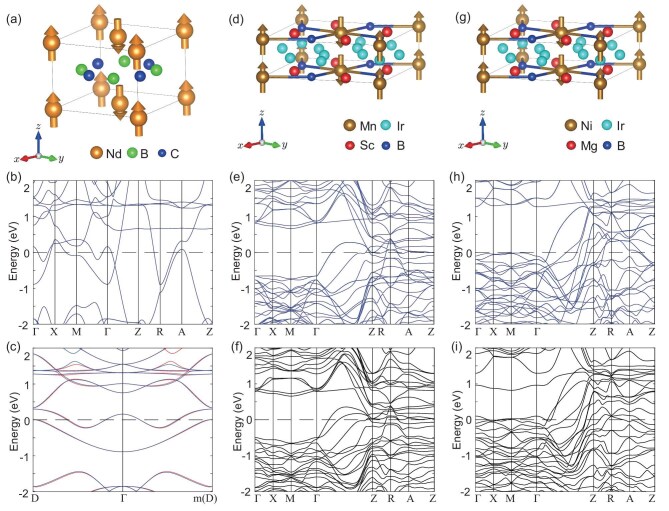
The crystal and electronic structures of altermagnets. (a) The NdB$_2$C$_2$ crystal primitive cell with magnetic structure. (b, c) The electronic band structure of altermagnetic NdB$_2$C$_2$. The electronic structure is calculated under the correlation interaction ${\rm U}= 5\, {\rm eV}$. (d) The Sc$_2$MnIr$_5$B$_2$ crystal primitive cell with magnetic structure. (e, f) The electronic band structure of altermagnetic Sc$_2$MnIr$_5$B$_2$ without and with SOC, respectively. The electronic structure is calculated under the correlation interaction $\rm {U= 4\, eV}$. (g) The Mg$_2$NiIr$_5$B$_2$ crystal primitive cell with magnetic structure. (h, i) The electronic band structure of altermagnetic Mg$_2$NiIr$_5$B$_2$ without and with SOC, respectively. The electronic structure is calculated under the correlation interaction $\rm {U= 6.56\, eV}$. The red and blue lines represent spin-up and spin-down energy bands, respectively.

Interestingly, the metallic altermagnet $\text{Nd}\text{B}_2\text{C}_2$ has odd-under-time-reversal Dirac fermions protected by the spin symmetries $\lbrace E||C_{4z}\rbrace$ and $\lbrace C_2^{\perp }||M_{x}(\frac{1}{2},\frac{1}{2})\rbrace$ (see Fig. 3(b)), but $\text{Sc}_2\text{Mn}\text{Ir}_5\text{B}_2$ and $\text{Mg}_2\text{Ni}\text{Ir}_5\text{B}_2$ have odd-under-time-reversal six-fold degenerate fermions (see panels (e) and (h) of Fig. 3) on the $\Gamma$-Z axis around the Fermi level, which is protected by the spin point group symmetry. When considering spin-orbit coupling (SOC), the three metallic altermagnets $\text{Nd}\text{B}_2\text{C}_2$, $\text{Sc}_2\text{Mn}\text{Ir}_5\text{B}_2$ and $\text{Mg}_2\text{Ni}\text{Ir}_5\text{B}_2$ have $D_{4h}$ point group symmetry that must be broken in ferromagnets, and the $C_{4v}$ double point group symmetry protects the odd-under-time-reversal Dirac fermions of the metallic altermagnets $\text{Sc}_2\text{Mn}\text{Ir}_5\text{B}_2$ and $\text{Mg}_2\text{Ni}\text{Ir}_5\text{B}_2$ on the $\Gamma$-Z axis (see panels (f) and (i) of Fig. 3). Moreover, the pair of odd-under-time-reversal Dirac points in $\text{Mg}_2\text{Ni}\text{Ir}_5\text{B}_2$ are very close to the Fermi level, which is an advantage for investigating its novel physical properties in experiments.

**Figure 4.**
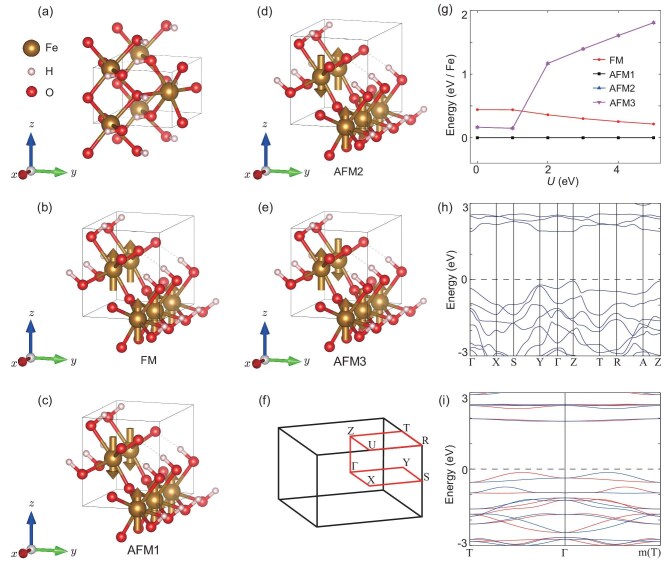
The crystal and electronic structures of altermagnet FeHO$_2$ (31). (a) The crystal primitive cell of altermagnetic FeHO$_2$ (31). (b–e) Four significant magnetic structures of FeHO$_2$ (31). The arrows represent the magnetic moments of Fe. (f) The Brillouin zone (BZ) with high-symmetry points and lines of altermagnetic FeHO$_2$ (31). (g) The relative energy of four significant magnetic states with the variation of correlation interaction U. (h, i) The electronic band structure of FeHO$_2$ (31) without SOC. The red and blue lines represent the spin-up and spin-down energy bands, respectively. The electronic structure is calculated under the correlation interaction $\rm {U= 4\, eV}$.

On the other hand, ferromagnetic semiconductors that have spintronic and transistor functionalities could be applied to the next generation of electronic devices. However, the ferromagnets are usually metals with a high Curie temperature and hold no brief for insulators with a high Curie temperature. Altermagnets with compensated antiparallel sublattices are not only in favor of insulators with a high Neel temperature, but also have spintronic functionality [3]. Thus, altermagnets open a new pathway to bypass the difficulties of ferromagnets. Here, we employed the LDA+U method [85] to predict 34 altermagnetic semiconductors (see Table S2). Furthermore, altermagnetic $\text{Fe}\text{H}\text{O}_2$ may be a spin-triplet excitonic phase. From Fig. 4(i), we observe that there is large spin splitting of 0.39 eV in the T-$\Gamma$-T directions and the spins of the valence and conduction band are opposite, which may result in the spin-triplet excitonic phase [86]. Moreover, the energy of the altermagnetic state (AFM1) is much lower than that of the other three magnetic states (see Fig. 4(g)), indicating that $\text{Fe}\text{H}\text{O}_2$ may have a Neel temperature above room temperature. Thus, altermagnetic $\text{Fe}\text{H}\text{O}_2$ is a very interesting material that, we believe, will attract both theoretical and experimental interest. In addition, although $\text{Nd}\text{Ru}\text{O}_3$ is an altermagnetic semimetal, it has a band gap along the high-symmetry directions with spin splitting, and its valence and conduction bands have opposite spins (see panels e and f of Fig. S5). Thus, $\text{Nd}\text{Ru}\text{O}_3$ may be a Bardeen–Cooper–Schrieffer-type triplet exciton insulator [87]. In the following, we present in detail two altermagnetic materials that are a metal and semiconductor, respectively.

**Figure 5.**
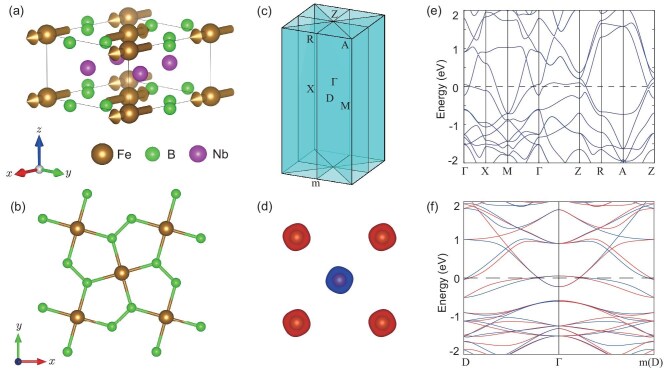
The crystal and electronic structures of altermagnetic $\text{Nb}_2\text{Fe}\text{B}_2$. (a) The side view of altermagnetic $\text{Nb}_2\text{Fe}\text{B}_2$. (b) The top view of altermagnetic $\text{Nb}_2\text{Fe}\text{B}_2$. (c) The BZ with high-symmetry points of altermagnetic $\text{Nb}_2\text{Fe}\text{B}_2$. The cyan plane represents the nodal surface of the BZ. Here m represents the mirror symmetry $M_y$. (d) The anisotropic spin-charge density deriving from an anisotropic crystal field. (e) The electronic band structure along high-symmetry directions without SOC. (f) The electronic band structure along non-high-symmetry directions without SOC. The red and blue lines represent the spin-up and spin-down energy bands, respectively. The electronic structure is calculated under the correlation interaction $\rm {U= 4.82\, eV}$.

Altermagnetic $\text{Nb}_2\text{Fe}\text{B}_2$ has space group $P4$/$mbm\, (127)$ symmetry, and the corresponding elementary symmetry operations are $C_{4z}, C_{2x} (\frac{1}{2}, \frac{1}{2})$ and *I*, which yield the point group $D_{4h}$. The crystal structure of $\text{Nb}_2\text{Fe}\text{B}_2$ is composed of an Fe-B atom layer and Nb atom layer, as shown in Fig. 5(a). Moreover, the two Fe atoms in the primitive cell are surrounded by two *B* atomic quadrilaterals with different orientations (Fig. 5(b)). Very recently, $\text{Nb}_2\text{Fe}\text{B}_2$ has been predicted to be a Neel antiferromagnet, which is shown in Fig. 5(a). Because of the anisotropic Fe-B quadrilateral, the spin-charge density of Fe atoms is anisotropic (see Fig. 5(d)). Thus, compensated antiparallel spins are not connected by the spin symmetry $\lbrace C_2^{\perp }||I\rbrace$ or $\lbrace C_2^{\perp }||\tau \rbrace$, but are connected by the spin symmetry $\lbrace C_2^{\perp }||C_{2x}(\frac{1}{2},\frac{1}{2})\rbrace$; that is, $\text{Nb}_2\text{Fe}\text{B}_2$ is an altermagnetic material. The spin symmetry $\lbrace C_2^{\perp }||M_{x}(\frac{1}{2},\frac{1}{2})\rbrace$ protects the spin degeneracy in electronic bands on the $k_x= 0$ and $\pi$ planes; considering the spin symmetries $\lbrace E||C_{4z}\rbrace$, altermagnetic $\text{Nb}_2\text{Fe}\text{B}_2$ has six node surfaces in the Brillouin zone (see Fig. 5(c)). Thus, $\text{Nb}_2\text{Fe}\text{B}_2$ is a *g*-wave altermagnet described by the non-trivial spin Laue group $P^14/^1m^2m^2m$. Figure 5(e) shows that the electronic bands of altermagnetic $\text{Nb}_2\text{Fe}\text{B}_2$ are spin degenerate along the high-symmetry directions, which is consistent with our symmetry analysis. As can be seen from Fig. 5(f), all the bands are spin splitting and spin antisymmetric in the non-high-symmetry D-$\Gamma$-m(D) direction, which reflects the characteristics of *g*-wave spin polarization. On the other hand, the valence bands and the conduction bands have multiple crossing points in the high-symmetry and non-high-symmetry directions, such as the $\Gamma$-X and $\Gamma$-D directions, indicating that altermagnet $\text{Nb}_2\text{Fe}\text{B}_2$ is a topologically non-trivial metal. When considering SOC, the easy magnetization axis of altermagnet $\text{Nb}_2\text{Fe}\text{B}_2$ is along the *x* direction. Accordingly, altermagnet $\text{Nb}_2\text{Fe}\text{B}_2$ has $C_{2z}T, C_{2x}(\frac{1}{2}, \frac{1}{2})T, C_{2y}(\frac{1}{2}, \frac{1}{2})T, I, M_zT, M_x(\frac{1}{2}$, $\frac{1}{2})T$, $M_y$ point symmetries, which make the anomalous Hall conductivities both $\sigma _{xy}$ and $\sigma _{yz}$ zero, but $\sigma _{xz}$ non-zero, which has been predicted by our previous theoretical study [84]. Likewise, the anomalous Kerr effects can also be realized in altermagnet $\text{Nb}_2\text{Fe}\text{B}_2$.

**Figure 6.**
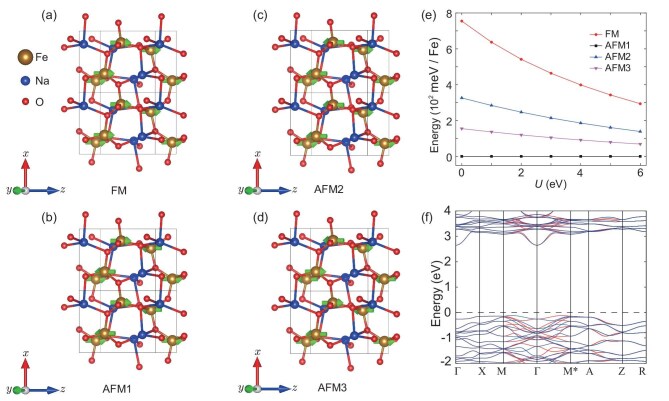
The crystal and electronic structures of altermagnetic $\text{Na}\text{Fe}\text{O}_2\, (92)$. (a–d) The crystal structures of $\text{Na}\text{Fe}\text{O}_2\, (92)$ with FM, AFM1, AFM2 and AFM3 structures. (e) Relative energy of different magnetic states with the variation of the correlation interaction U. (f) The electronic band structure of $\text{Na}\text{Fe}\text{O}_2\, (92)$ along high-symmetry directions without SOC. The red and blue lines represent the spin-up and spin-down energy bands, respectively. The electronic structure is calculated under the correlation interaction $\rm {U= 4\, eV}$.

The other altermagnetic material that we would like to mention is $\text{Na}\text{Fe}\text{O}_2$. The crystal structure of $\text{Na}\text{Fe}\text{O}_2$ is shown in panels (a)–(d) of Fig. 6 with space group $P/4_12_12\, (92)$ symmetry. The corresponding elementary symmetry operations are $C_{4z} (\frac{1}{2}, \frac{1}{2}, \frac{1}{4})$ and $C_{2x} (\frac{1}{2}, \frac{1}{2}, \frac{3}{4})$, which yield the point group $D_4$. Since the *d* orbitals of Fe are half occupied and the angle between Fe-O-Fe is 136$^\circ$ in $\text{Na}\text{Fe}\text{O}_2$, the superexchange interactions result in the nearest-neighbor Fe ions having opposite magnetic moments and the next-neighbor Fe ions having the same magnetic moments. Hence, the magnetic ground state of $\text{Na}\text{Fe}\text{O}_2$ will be AFM1 (see Fig. 6(b)). To verify our theoretical analysis, we consider four different magnetic structures, which are shown in panels (a)–(d) of Fig. 6. It can be seen that the magnetic structure AFM1 is always in the ground state of $\text{Na}\text{Fe}\text{O}_2$ under different correlation interactions U (see Fig. 6(e)). Moreover, the energy of the AFM1 state is much lower than that of the other three magnetic states (Fig. 6(e)), implying that $\text{Na}\text{Fe}\text{O}_2$ may have a Neel temperature above room temperature. We show in Fig. 6(b) that the magnetic and crystal primitive cells of $\text{Na}\text{Fe}\text{O}_2$ are the same, which break $\lbrace C_2^{\perp }||\tau \rbrace$ spin symmetry. Thus, $\text{Na}\text{Fe}\text{O}_2$ is an altermagnetic material due to the lack of space-inversion symmetry.

We also calculated the electronic band structure along the high-symmetry directions. Figure 6(f) shows that altermagnet $\text{Na}\text{Fe}\text{O}_2$ is a semiconductor with a band gap of 2.75 eV. The spin-degenerate bands in the $\Gamma$-${\rm {X,\, M}}^{*}$-${\rm {A}}$ and $\rm {Z}$-$\rm {R}$ directions (the $\rm {X}$-$\rm {M}$ direction) are protected by the spin symmetry $\lbrace C_2^{\perp }||C_{2y} (\frac{1}{2}, \frac{1}{2}, \frac{1}{4})\rbrace$ ($\lbrace C_2^{\perp }||C_{2y} (\frac{1}{2}, \frac{1}{2}, \frac{3}{4})\rbrace$); see Fig. 6(f). In fact, the spin symmetry $\lbrace T||C_{2y}T (\frac{1}{2}, \frac{1}{2}, \frac{1}{4})\rbrace$ ($\lbrace T||C_{2x}T (\frac{1}{2}, \frac{1}{2}, \frac{3}{4})\rbrace$) can protect spin degeneracy of bands on the $k_y =0$ and $\pi$ (the $k_x = 0$ and $\pi$) planes. That is, altermagnet $\text{Na}\text{Fe}\text{O}_2$ has four nodal surfaces in the Brillouin zone. Thus, $\text{Na}\text{Fe}\text{O}_2$ is a *d*-wave altermagnet that is reflected by the spin-splitting bands in the $\rm {M}$-$\Gamma$-$\rm {M}^{*}$ directions. Considering the *d*-wave altermagnets allowing unique spin current by electrical means [5], altermagnet $\text{Na}\text{Fe}\text{O}_2$ may have both spintronic and transistor functionalities at room temperature.

## DISCUSSION

AI approaches have shown ground-breaking capabilities in the discovery of materials in a large search space. An intractable challenge faced by AI lies in the shortage of sufficient labels or positive samples, e.g. in the case of the discovery of altermagnetic materials. We herein introduced an AI search engine that combines pre-trained crystal models (GNN pre-training and optimal transport theory) and physics-based methods (symmetry analysis and first-principles electronic structure calculations) to discover new altermagnetic materials with specific properties under minimal labeled sample conditions. Among the 91 649 possible candidates, we identified 50 new altermagnetic materials covering metals, semiconductors and insulators. Meanwhile, the proposed AI search engine also has the few-shot learning ability. For example, it is capable of predicting 25 altermagnetic materials only based on 14 positive samples (see Note C within the online supplementary material). We observed various novel physical properties in these newly discovered altermagnetic materials, e.g. the anomalous Hall effect, anomalous Kerr effect and topological property. It is noted that four out of these 50 altermagnetic materials are *i*-wave types, discovered for the first time, filling a gap in the literature. We demonstrate that the AI search engine is capable of uncovering a set of altermagnetic materials with unique properties, highlighting its potential for accelerated discovery of the materials with targeting properties.

There still remain some potential limitations associated with the AI search engine. Firstly, we have to admit that the issue of imbalance between positive and negative samples during the fine-tuning stage exists, primarily due to the scarcity of known positive samples. We have also discussed the potential intrinsic error and computational cost associated with this AI search engine (see Note C within the online supplementary material). Utilizing the translational and rotational symmetries of crystals to augment positive sample data may help address this challenge, which will be demonstrated in our future work. Secondly, if the materials exhibit distinct magnetic phases at varying temperatures, such as Mn$_5$Si$_3$, whose low-temperature spin pattern is reported to be non-collinear and high-temperature spin pattern to be antiferromagnetic [88], our current model is unable to predict their magnetic properties. In such a case, if the material with temperature-driven magnetic transition is used as the positive sample to train the AI model, the prediction might involve possible bias. We improve our classifier model by considering temperature as a conditional input parameter to enhance its capability in screening and predicting materials with temperature-driven properties in the future. Thirdly, in the calculations of magnetic ground states, we focus only on collinear magnetic structures and do not consider non-collinear ones. This is because most of the materials we calculate do not exhibit geometric frustration. Even for a few materials like CoF$_3$, which exhibit triangular geometric frustration, previous studies did not consider non-collinear magnetic structures [56]. Another limitation is that we have not yet found ideal altermagnetic topological insulators and altermagnetic topological semimetals (such as odd-under-time-reversal Dirac semimetals, and six-fold semimetals). Employing the decoder based on the pre-trained model to generate potential altermagnetic materials holds promise in overcoming this challenge. Furthermore, adopting a multimodal pre-training approach offers the potential to further enhance the accuracy of model predictions. The current pre-training only considers the single modality of the crystal structure information. Leveraging information from other modalities (such as textual descriptions of crystal structures) may enhance the performance of the pre-trained model. These methods will be further explored in our future research endeavors.

In addition, an alternative to alter the proposed AI model is to replace the classifier with a regressor to predict the magnetic structure of a given material candidate, where such a regressor can be fine-tuned based on 2138 known magnetic structures in the MAGNDATA database [28]. Afterward, symmetry analysis can be employed to identify the altermagnetism. However, since there are infinite types of magnetic structures, accurately predicting the exact type of magnetic structure for a given material remains challenging. Therefore, an end-to-end classifier in our proposed model, to directly judge whether a crystal material is altermagnetic, is preferred, especially under the condition of very limited positive samples.

It is well known in the community that the altermagnetism of 98% of candidates in the Materials Project ($>\!90\, 000$ materials), whose magnetic structure information is unknown, has not yet been confirmed and remains a substantially challenging task. The brute-force approach leveraging our expert knowledge relies on trial and error by chance, having an extremely low probability of correctly discovering and confirming altermagnetic materials from the large database. However, our AI model narrows down the search space, predicts a list of highly possible candidate altermagnets and lifts the discovery accuracy to a notable margin of about 31% (50/161), which greatly accelerates our discovery of new altermagnetic materials. (If we calculate the magnetic ground state of all materials, and then determine whether these materials are altermagnetic by spin symmetry, we can probably predict more altermagnetic materials. However, this cost is extremely huge. Given the scale and complexity of this task, it is unlikely that DFT can be used to complete the above brute-force computation for all candidate materials.) The success of this engine lies not only in its predictive capabilities, but also in its ability to leverage extensive crystal structure data and deep learning techniques, allowing for pre-training without explicit reference to underlying physical laws, to reveal complex correlations and patterns in new materials. Although 161 altermagnetic materials have been confirmed by the symmetry analysis approach [23], the urgency of discovering a variety of new altermagnetic materials with different properties still remains. Based on these existing altermagnetic materials confirmed by the spin space group, our AI search engine could predict many more altermagnetic materials, among which we expect to find a variety of altermagnets beyond the MAGNDATA database. For example, over 300 additional candidate materials (unconfirmed yet by DFT calculations) were predicted by the AI search engine and listed in our GitHub repository (https://github.com/zfgao66/MatAltMag) for open research. Nevertheless, obtaining the magnetic ground state without experimental validation remains a challenging problem. The magnetic state of a material may be collinear or non-collinear, and the magnetic cell may be a supercell of the crystal primitive cell, which, in principle, lead to an infinite number of possibilities for the magnetic structure. Hence, experimental efforts will help further validate our discovery.

The proposed engine might also be applicable to other types of materials whose properties are strongly correlated to their crystal structures, such as Bardeen–Cooper–Schrieffer superconductive materials [89], the ferromagnetic semiconductor [90] and high-temperature superconductive materials [91], among others. We will demonstrate the potential of our proposed model for discovering other materials in a future study. We envision this effort may present new opportunities in the field of material discovery across different disciplines.

## METHODS

We herein introduce the model details and implementation specifics.

### Model details

#### Architecture overview

The concept of pre-training a large deep learning model and subsequently applying it to perform downstream tasks originally originated in the field of natural language processing (NLP). Large-scale NLP models, such as GPT [92], and their derivatives, employ transformers as text encoders. These encoders transform input texts into embeddings and establish pre-training objectives based on these embeddings, including generative loss and masked language modeling loss. The pre-training process is typically unsupervised, based on large-scale unlabeled samples. In contrast to traditional end-to-end neural network models, pre-trained models can achieve excellent performance even with limited labeled positive samples. We thus consider utilizing the pre-training technique to fully leverage the information from existing crystal material databases and treat the discovery of altermagnetic materials as a downstream task.

The objective of our proposed pre-training model for crystal materials is to learn the information embedded within crystal structures. To enhance the learning capacity of the pre-training model, we proposed a graph auto-encoder architecture (see Figs 1 and 2). The encoder consists of *n* layers of graph convolution to learn crystal embeddings, while the decoder employs the Wasserstein distance based on optimal transport theory [40] for reconstruction of the input crystal structures. Specifically, the encoder aims to encode the graphical representation of crystal materials into a high-dimensional matrix, while the goal of the decoder is to decode this one back into the graphical representation of crystal materials. Through extensive training with unlabeled data, the model effectively converges (as depicted in the pre-training loss history shown in Fig. S3). We believe that the pre-trained encoder can effectively project the crystal structures into crystal embeddings. Leveraging the encoder of the pre-trained model, we built the classifier model by incorporating a pooling layer and a softmax function. Subsequently, we trained the classifier model using the fine-tuning dataset. This trained model is then employed to screen the candidate materials, offering the probability of whether the target material is altermagnetic. The hyperparameters of the model were obtained by grid search, as listed in Table S1. In summary, our model comprises four main components: crystal data pre-processing, an encoder constructed using graph convolutional neural networks, a decoder built based on optimal transport theory and the construction of a classifier model. We elaborate on each of these components one by one as follows.

#### Crystal data pre-processing

The data pre-processing procedure aims to bridge the crystal structure and the crystal graph representation [37]. The input of the model is a crystal structure file (.cif) that contains three primitive translation vectors of the primitive unit cell and the positional information of each atom. It satisfies the organization invariance for atomic indexing and the size invariance for unit cell selection. We define the graph representation $\mathcal {G}(V, U, X)$ to describe the crystal structure information, where *V* denotes the set of nodes, *U* the set of edges and *X* the set of features. First, we represent atoms as nodes $v_i$ in a crystal graph representation, where $i=1,\dots , |V|$. Since periodic boundary conditions are taken into consideration, equivalent nodes are merged to obtain irreducible nodes. Then, for each node $v_i$, we consider the neighborhood nodes $v_j$, where $j=,1,\dots ,|\mathcal {N}_i|$ and $\mathcal {N}_i$ is the set of neighborhood nodes for $v_i$. The *k* connections between nodes $v_i$ and $v_j$ are denoted as the edge $u_{(i,j)_k}$ in the graph. Next, the initial node features $\lbrace h_{i}^{(0)}\rbrace _{i=1}^{|V|}$ are given through one-hot encoding based on the sequence of atoms in the crystal structure. We use $H_{\mathcal {N}_i}^{(0)}$ to denote the neighbor node features of node $v_i$. Here, $v_i$ denotes the *i*th node. Each edge $u_{(i,j)_k}\in U$ is represented by a feature vector $u_{(i,j)_k}$ that corresponds to the *k*th bond linking node $v_i$ and node $v_j$. A feature vector $h_i\in X$ encoding the attribute of the atom corresponding to node $v_i\in V$ is used to represent each node $v_i$. An example for determining the atom connectivity is illustrated in Fig. S4.

#### Crystal graph convolutional encoder

The encoder is used to represent the input crystal structure information as a high-dimensional matrix (Fig. 2(c)), which contains *n* convolutional layers. The *t*th convolutional layer updates the node feature vector $h_i^{(t)}$ via the convolution function $h_i^{(t+1)}={\rm {Conv}}(h_i^{(t)},h_j^{(t)},u_{(i,j)_k})$. We denote the graph convolution function by *g*, which iteratively updates the overall feature vector $h_i$, whose output is the input for the next step. The node index in feature vector $h_i$ and length of $h_i$ are invariant for every step. We construct the first concatenate neighbor vector as $z^{(t)}_{(i,j)_k}=h^{(t)}_i\oplus h^{(t)}_j \oplus u_{(i,j)_k}$ in step *t*, and then perform the convolution operation to update the feature as


(1) \begin{eqnarray*}
h_i^{(t+1)}=h_i^{(t)}+ \sum _{v_j\in \mathcal {N}_i,\, v_m\in \mathcal {M}_i,k}\sigma (A)\odot g(B),
\end{eqnarray*}

where *A* denotes $z^{(t)}_{(i,j)_k} W_f^{(t)}+b_f^{(t)}$ and *B* denotes $z^{(t)}_{(i,j)_k} W_s^{(t)}+h_{i,m}^{(t)}W_m^{(t)}+b_s^{(t)}$. Here ‘$\odot$’ denotes the elementwise multiplication, the $\mathcal {M}_i$ are the magnetic atoms corresponding to node $v_i$ and $\sigma$ is the sigmoid activation function. Since the magnetic atoms are important for the material to exhibit altermagnetic properties, we added the weight term $W_m^{(t)}$ for the magnetic atoms. The weight functions $W_c^{(t)}, W_s^{(t)}, W_m^{(t)}$ are the convolution weight matrix, self-weight matrix and magnetic atom weight matrix of the *t*th layer, respectively. In equation (1), we incorporate the residual term $h_i^{(t)}$ to enhance the training of the neural network.

#### Neighborhood Wasserstein reconstruction decoder

The decoder (denoted by $\psi$) is utilized to restore the input graph representation of a crystal from the crystal embeddings, which mainly consists of two parts (see Fig. 2(a)), one for node feature reconstruction (denoted by $\psi _s$) and the other for adjacent node feature reconstruction (denoted by $\psi _p$), namely, $\psi =(\psi _p+\psi _s)$. Here, $\psi _s={\rm {MLP}}_s(h_{i}^{(t)})$ is used to reconstruct the node features, where MLP indicates a multilayer perception. The architecture of the decoder block, as shown in Fig. 2(b), follows the design in [50].

In particular, we adopt the *n*-hop neighboring Wasserstein decoder for graph feature reconstruction. We can obtain $\lbrace h_i^{(0)},H_{\mathcal {N}_i}^{(0)}\rbrace$ from the pre-processing procedure. For each node $v_i \in V$, we update the node representation $h_i^{(t+1)}$ via the GNN layer in the encoder, which gathers information from $h_i^{(t)}$ and its neighbor representations $H_{\mathcal {N}_i}^{(t)}$, namely, $h_i^{(t+1)} = \phi ^{(t)} (h_i^{(t)},H_{\mathcal {N}_i}^{(t)})$. Note that the neighborhood set of node features $H_{\mathcal {N}_i}^{(t)}$ can be directly assembled based on the node adjacency. Consequently, we solve the following optimization problem to train the network:


(2) \begin{eqnarray*}
{\rm arg\, min}_{\phi ,\psi }\sum _{v_i \in V}\mathcal {L} \left(h_{i}^{(t)}, H_{\mathcal {N}_i}^{(t)}, \psi \left(h_i^{(t+1)}, H_{\mathcal {N}_i}^{(t+1)}\right)\!\right)\!.\!\!\!\!\! \\
\end{eqnarray*}

Here $\mathcal {L}(\cdot ,\cdot )$ denotes the reconstruction loss over $0\le t< n$. The loss function $\mathcal {L}$ can be decomposed into two distinct elements, each gauging the reconstruction of self and neighborhood node features, respectively, written as


(3) \begin{eqnarray*}
\mathcal {L}&=& \lambda _s\mathcal {L}_{s}\left(h_{i}^{(0)},\psi _s\left(h_{i}^{(n)}\right)\right)\\
&&+\, \lambda _p\sum _{t=0}^{n-1}\mathcal {L}_{p}\left(H^{(t)}_{Q_i}, \hat{H}^{(t)}_{Q_i}\right),\\
\end{eqnarray*}

where $\hat{H}^{(t)}_{Q_i} = \psi _p^{(t+1)}(h_{i}^{(t+1)}, H_{\mathcal {N}_i}^{(t+1)})$ denotes the reconstructed neighborhood set of node features based on the sampling network shown in Fig. 2(b). Here $Q_i\subset \mathcal {N}_{i}$ denotes the set of *q* samples of neighborhood nodes for $v_i$; $\lambda _s$ and $\lambda _p$ are the weighting coefficients and $\mathcal {L}_s$ stands for the reconstruction error of the node features, given by


(4) \begin{eqnarray*}
\mathcal {L}_{s}\left(h_{i}^{(0)},\psi _s\left(h_{i}^{(n)}\right)\right)=\left\Vert h_{i}^{(0)} -\psi _s\left(h_{i}^{(n)}\right)\right\Vert ^2_2.
\end{eqnarray*}

In equation (3), $\mathcal {L}_p$ is the loss function used to measure the reconstruction of the neighborhood set of node features $H^{(t)}_{Q_i}$. Inspired by Tang *et al.* [50], we evaluate this loss function by a Monte Carlo method. Specifically, for node $v_i$, the distribution of its neighborhood information can be empirically represented by $\mathcal {P}^{(t)}_{i}$, defined as


(5) \begin{eqnarray*}
\mathcal {P}^{(t)}_{i} = \sum _{v_j \in \mathcal {N}_{i}}\delta _{h_{j}}^{(t)},
\end{eqnarray*}

where $\delta _{h_{j}}^{(t)}$ denotes the Dirac delta function. Here, we adopt the 2-Wasserstein distance, which measures the similarity between two distributions, to construct the loss [50], expressed as


(6) \begin{eqnarray*}
\mathcal {L}_{p}\left(H_{\mathcal {N}_{i}}^{(t)}, \hat{H}^{(t)}_{Q_i}\right)= \mathcal {W}_2^2\left(\mathcal {P}^{(t)}_{i}, \hat{H}^{(t)}_{Q_i}\right).
\end{eqnarray*}

In our experiments, we fix $q=10$ based on a Hungarian matching, which avoids heavy computational overhead while retaining accuracy, when evaluating equation (6) during training.

#### Classifier model

The classifier model is constructed by adding a pooling layer and a softmax module after the encoder of the pre-trained model (see Fig. 2(c)). The pooling layer is applied to the embedding of the pre-trained encoder to generate an overall feature vector $h_g$ that can be represented by a pooling function given by $h_g={\rm {Pool}}(h_0^{(0)},h_1^{(0)},\dots ,h_N^{(0)},\dots , h_N^{(n)})$, where *n* is the number of convolution layers and *N* is the number of nodes in the graph. The softmax module in the classifier model ensures that the output for each candidate material through the model is a probability in the range $[0, 1]$, representing the likelihood of the candidate material being an altermagnetic material.

### Implementation details

#### The pre-training model

To extract the crystal embeddings of the candidate materials, we employ a graph convolution neural network as an encoder, which consists of three graph convolution layers. (We use the code of CGCNN to construct the encoder module [37] to construct the encoder module, whose GitHub link is https://github.com/txie-93/cgcnn and commit ID is ‘f42ab233c4ee0c416879d6bc2d22a264418413ad’.) For classification, we utilize a pooling layer and a multilayer perceptron as the projection head, comprising two fully connected layers with a rectified linear unit activation layer and a dropout layer. (We use the code of NWR-GAE to construct the auto-decoder module [50] to construct the auto-decoder module and compute loss, whose GitHub link is https://github.com/mtang724/NWR-GAE and commit ID is ‘e9aee57a009f6c2150cf68fc173c2af3094a7205’.) In terms of optimization, we use the Adam optimizer with a learning rate of 0.001. Additionally, we implement MultiStepLR with milestones set at 100. To ensure stability during training, we train our classifier model with a dropout rate of 0.25. This is done using a batch size of 512 and training over 500 epochs on two NVIDIA A100 GPUs. We then label these materials whose probabilities exceed 0.9 as potential altermagnetic candidates in all experiments. To train and evaluate our model efficiently, we leverage the distributed deep learning framework Accelerate. More details of the model training are discussed in Note A within the online supplementary material. Given this specific network architecture, we can complete the training and evaluation process of the classifier model in less than 1.5 hours on our datasets. This significantly improves the overall efficiency of our proposed workflow.

#### First-principles electronic calculation

The first-principles electronic structure calculations were performed in the framework of DFT using the Vienna ab initio simulation package (VASP) [93]. The generalized gradient approximation (GGA) of Perdew–Burke–Ernzerhof type was adopted for the exchange-correlation functional [94]. The projector-augmented-wave method was employed to describe the interactions between valence electrons and nuclei [95]. To account for the correlation effects of 3d and 4f orbitals, we performed GGA+U calculations by using the simplified rotationally invariant version introduced by Dudarev *et al.* [85]. Detailed information regarding the DFT calculations is given in Note D within the online supplementary material.

## Supplementary Material

nwaf066_Supplemental_File

## Data Availability

The crystal data are available from the Materials Project database via the web interface at https://materialsproject.org or the API at https://api.materialsproject.org. All source codes to reproduce the results in this study are available from GitHub (https://github.com/zfgao66/MatAltMag). We rely on PyTorch (https://pytorch.org) for deep model training. We use specialized tools for VASP (https://www.vasp.at/). The code of the pre-training model for crystal materials and the pre-trained neural network weights are available from GitHub (https://github.com/zfgao66/MatAltMag).
