# Supplementary material for: AI-accelerated discovery of altermagnetic materials
Source: Natl Sci Rev. 2025 Feb 22;12(4):nwaf066. doi: 10.1093/nsr/nwaf066 (PMC11983696; doi:10.1093/nsr/nwaf066)
Supplement: nwaf066_Supplemental_File [file nwaf066_supplemental_file.pdf]

Supplementary Information for:
AI-accelerated Discovery of Altermagnetic Materials

Ze-Feng Gao[1,2,†], Shuai Qu[2,†], Bocheng Zeng[1,†], Yang Liu[3], Ji-Rong Wen[1], Hao Sun[1,*], Peng-Jie Guo[2,*], Zhong-Yi Lu[2,*]

[1]Gaoling School of Artificial Intelligence, Renmin University of China, Beijing, China
[2]Department of Physics, Renmin University of China, Beijing, China
[3]School of Engineering Science, University of Chinese Academy of Sciences, Beijing, China

[†]Equally contributed

[*]Corresponding authors

Contents

A Addition information for pre-trained model **1**

B Addition altermagnetic materials confirmed by electronic structure calculations **3**

C Addition discussion for proposed AI search engine **20**

D Addition information for DFT calculations **21**

This supplementary document provides a detailed description of the proposed pre-trained model, dataset statistics, hyperparameter value, and details of altermagnetic materials confirmed by electronic structure calculations.

A Addition information for pre-trained model

Model architecture. We have two models in total, which are the auto-encoder model and the classifier model. Fig. 2 in the Main text shows the overall architecture and details of models. We pre-process the crystals and construct a graph for each crystal according to its structure and atoms. In Fig. 2 in the Main text, $\boldsymbol{\mu}_v$ stands for features from the crystal graph.

Workflow for symmetry analysis. Fig. S.1 has shown the workflow for symmetry analysis, through this screening process, we can build the pre-training dataset, fine-tuning dataset, and candidate materials dataset.

Visulization of the learned crystal embeddings. We perform visualization for the learned crystal embeddings of the candidate materials dataset, which we projected into 20 principal components with the PCA technique. We present the pair-wise visualization of three or two selected principal components of the learned crystal embeddings. Moreover, we also perform the T-SNE procedure to plot the 20 principal components to reveal the obvious clustering phenomena.

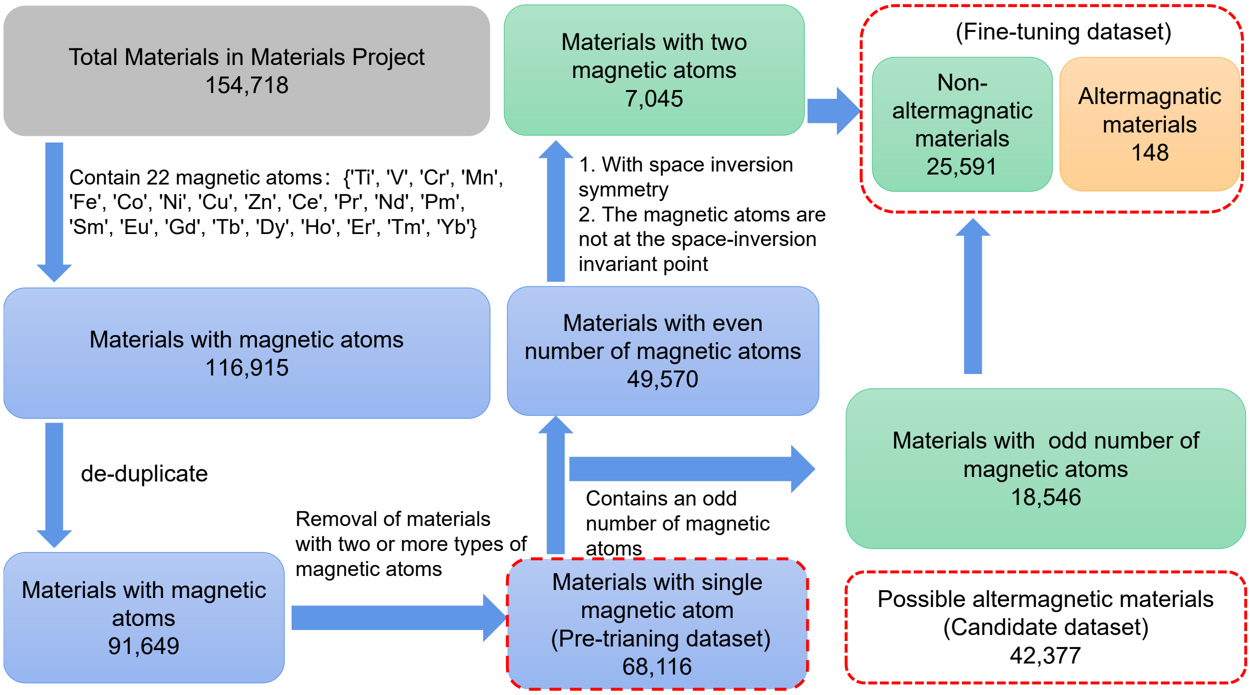

Figure S.1: The workflow for screening altermagnetic materials based on symmetry analysis. Through this screening process, we can build datasets for pre-training and fine-tuning, as well as a candidate set of possible altermagnetic materials.

Learning curve of auto-encoder. We pre-train our auto-encoder model for only 10 epochs. To show the changing trend of pre-train loss, we choose the number of batches as the x-coordinate instead of epochs in Fig. S.3.

Details for pre-processing. The 3-dim crystal structure is straightforward and intuitive, but it is awkward to deal with in code. Thus, the crystal graph arises from the 3-dim crystal structure. The part of negligible information is not significant, such as the weak bonds. The nodes and edges represent the atoms and bonds, respectively. The nodes are connected with edges. We still retain the important information of atoms and bonds. The magnetic atoms are extracted with the corresponding weight function. We are concerned with the material information of altermagnetic, which is critical and indispensable.

The feature vector h is used throughout each step. In order to make it easier to understand, we are going to expand on it. The feature vector h is analogous to the occupation number representation in quantum mechanics with the range from unoccupied 0 to occupied 1. The occupancy probability given by the subsequent iterations is still between 0 and 1 and it is the superposition of the wannier wave functions of the unoccupied state and occupied state. The element order information in the feature vector h is read from the structure file, and the order is fixed. There is also a significant eigenvector z, which is effective in complementing the information about the interaction strength of the bonds. The initial eigenvector $u(i,j)_k$ has only two values of 0 and 1, without bond 0 and with bond 1. However, the strong and weak information will be manifested by the relative magnitude with the range from 0 and 1 in the subsequent iterations. The stronger bonds will be close to 1, and the weaker bonds will be close to 0. An example of pre-processing for FeB to graph representation is shown in Fig. S.4.

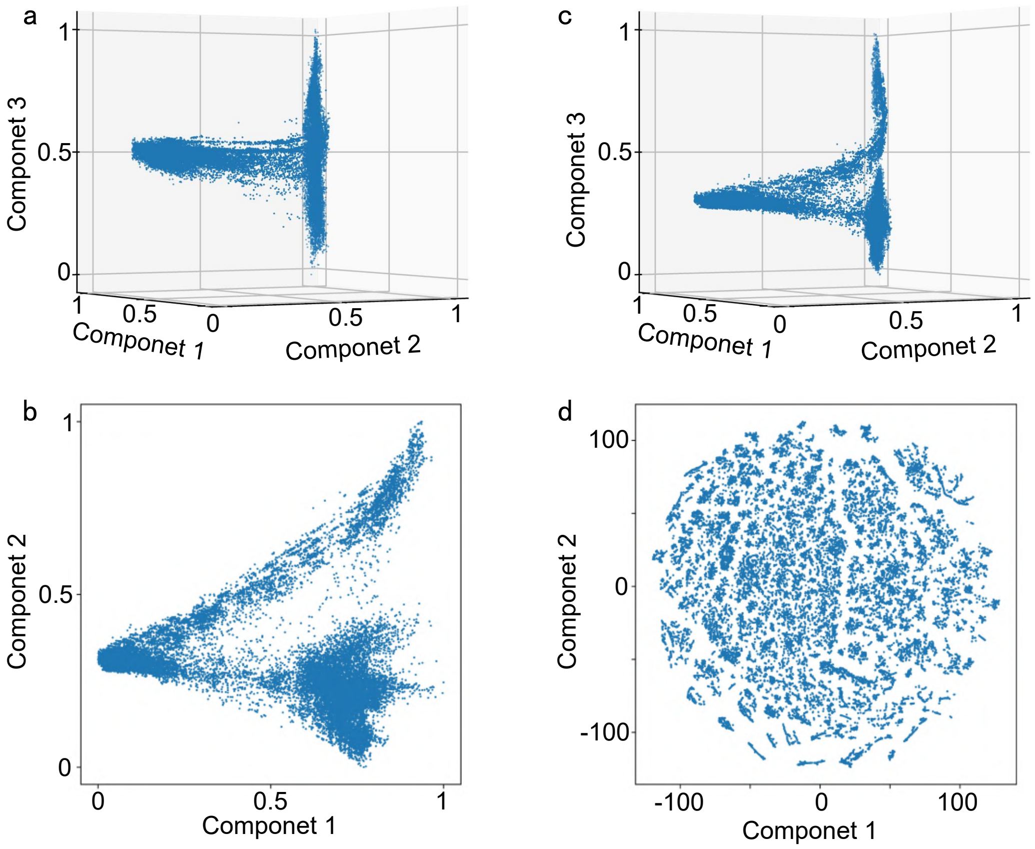

Figure S.2: Visualization of the learned crystal embeddings of the candidate materials, projected into 20 principal components by PCA. a–c, Pair-wise visualization of three or two selected principal components of the learned crystal embeddings. **d**, The t-distributed stochastic neighbor embedding (t-SNE) plot of the 20 principal components, reveals obvious clustering phenomena.

Hyperparameter value. In the pre-processing procedure, the crystal structure files (.cif) are transformed into the crystal graph data which can be fed into the model directly. Nodes of the graph are represented by atoms naturally and the atoms will be regarded as the neighbors of another atom if they are in the unit cell, out to a distance of radius. In the pre-training procedure, using the pre-training dataset consisting of 68,116 materials, we pre-train our auto-encoder model for 10 epochs with a batch size of 64 on 2 NVIDIA A100 GPUs, which takes about 2 days. Once pre-training is over, we load the weights of the encoder module into the classifier model and begin training for 500 epochs with a batch size of 512 and a drop rate of 0.25. The learning rate of both models is 0.001 and the hidden dimension of the encoder module is 512.

B Addition altermagnetic materials confirmed by electronic structure calculations

For the 19 materials ($NdRuO_3$, $NaFeO_2$(31), MnO_2(62), MnO_2(87), $CaLaCr_2O_6$, $Ca_3Cr_2O_7$, $CaLaFeAgO_6$, $ZrCrO_3$, $ZrMnO_3$, VF_3, CrF_3, NiF_3, $CaMnN_2$, $Ir_5B_2Mg_2Fe$, $Ir_5B_2Mg_2Mn$, $Ir_5B_2Mg_2Ni$, $Ir_5B_2Sc_2V$, $Ir_5B_2Sc_2Mn$), we determine their magnetic ground states by calculating the energies of their different magnetic structures under different correlated interactions U. Then, symmetry anal-

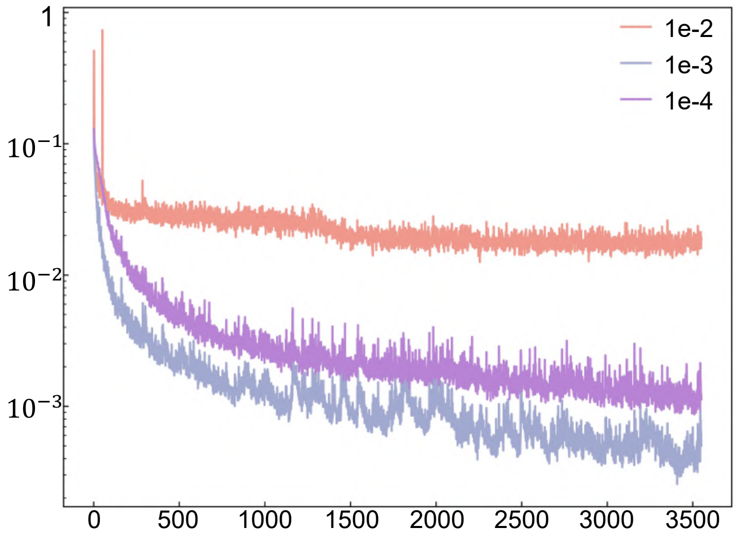

Figure S.3: Learning curve of the auto-encoder model with varying learning rate. The x-coordinate is the number of batches while the y-coordinate is the MSE loss.

Table S.1: Hyperparameter value obtained by grid search.

Hyperparameter	Auto-encoder	Classifier	Description
Epochs	10	500	Training epoch
Learning rate	1.0e-3	1.0e-3	Learning rate for optimizing neural network
Batch size	64	512	Number of input token per batch
Hidden dimension	512	512	Size of dimensions in the graph convolution layer
Sample size	10	-	Number of samples during the reconstruction
Radius	20	20	Neighbor distance of crystal atoms
Drop rate	-	0.25	Dropout rate for Classifier

ysis is used to determine that these magnetic ground states are altermagnetic states. Furthermore, symmetry analysis is also used to determine whether these altermagnetic materials are d-wave, g-wave, or i-wave. Finally, the electronic band structures are used to demonstrate our symmetry analysis. All calculated results are shown in Figs. S.5–S.17.

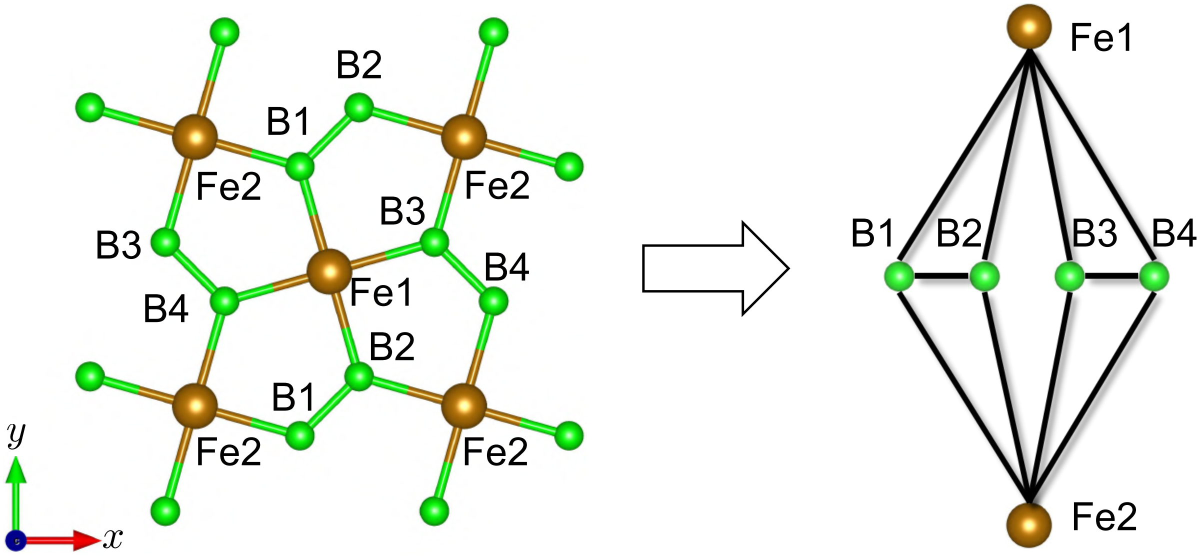

Figure S.4: An example of pre-processing for crystal structure to a crystal graph representation. The green nodes denote the B atoms, and the brown nodes denote the Fe atoms. The Fe1 node connects B1, B2, B3, and B4 nodes by 4 edges. It is pronounced that the Fe2 node connects the B1 and B4 nodes. The Fe nodes are consistent with each other in the 4 corners with periodic boundary conditions. Thus, the Fe2 node connects B1, B2, B3, and B4 nodes and the Fe1 node. Furthermore, the top B2 node connects to the bottom B1 node, and the left B3 node connects to the right B4 node.

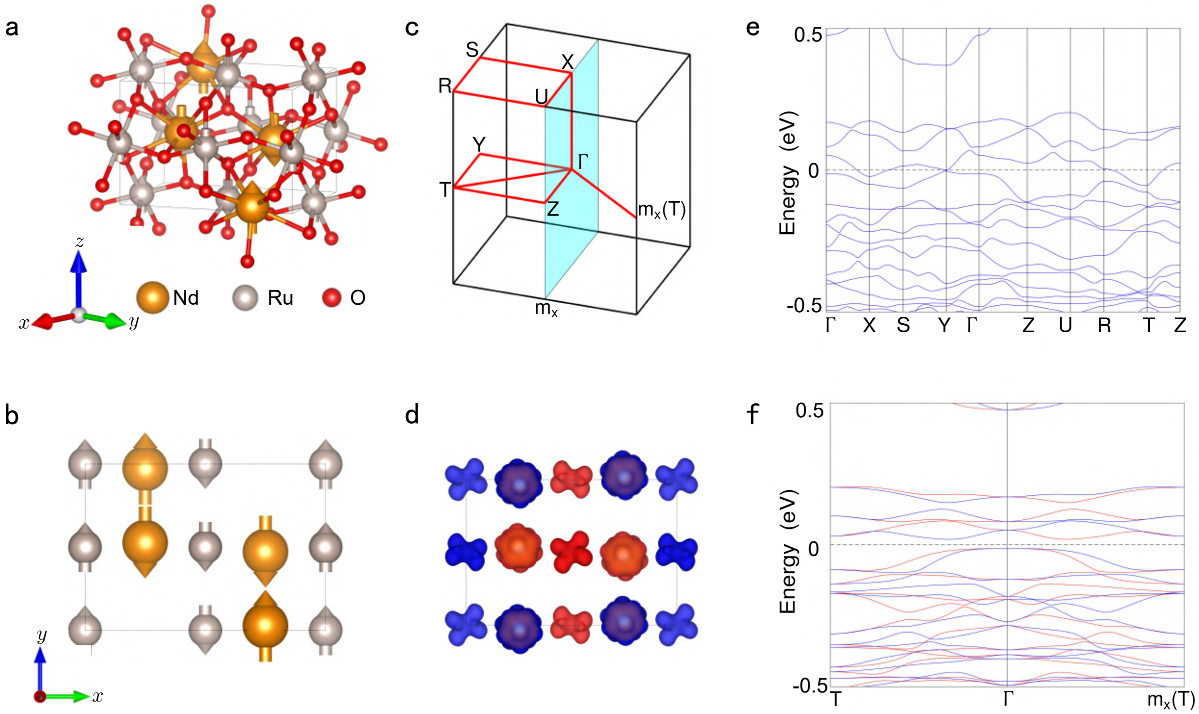

Figure S.5: The crystal and electronic structure of the altermagnet $NdRuO_3$. **a**, The side view of the magnetic primitive cell of the altermagnetic $NdRuO_3$. **b**, The top view of the magnetic primitive cell of the altermagnetic $NdRuO_3$. The arrows represent the magnetic moments of Nd and Ru. **c**, The Brillouin zone (BZ) with high-symmetry points and lines of altermagnetic $NdRuO_3$. **d**, The anisotropic spin-charge density deriving from an anisotropic crystal field. **e** and **f** are the electronic band structure of $NdRuO_3$ without SOC along different high-symmetry directions. The red and blue lines represent the spin-up and spin-down energy bands, respectively. The electronic structure is calculated under correlation interaction U = 7 eV and U = 2 eV for 4f orbits of Nd and 4d obits of Ru.

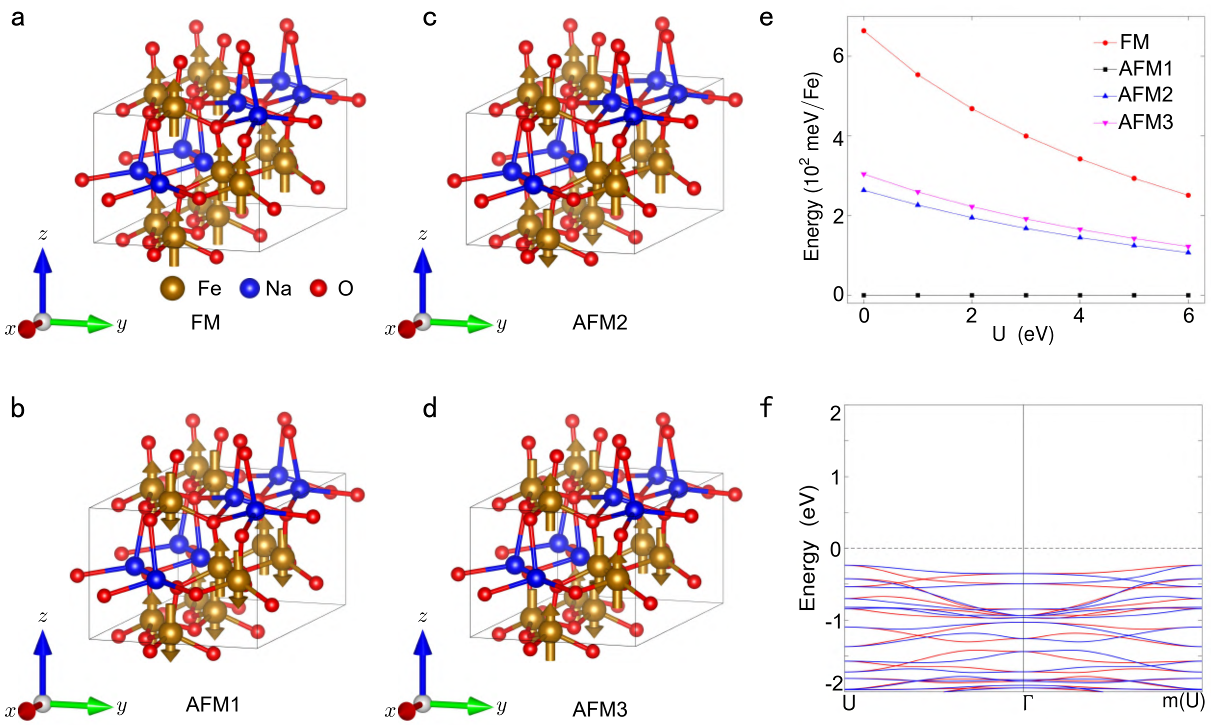

Figure S.6: Altermagnetic $NaFeO_2$(33) is confirmed by electronic structure calculations. **a**, The crystal structures of the $NaFeO_2$(33) with FM. **b**, The crystal structures of the $NaFeO_2$(33) with AFM1. **c**, The crystal structures of the $NaFeO_2$(33) with AFM2. **d**, The crystal structures of the $NaFeO_2$(33) with AFM3. The arrows represent the magnetic moments of Fe. **e**, The relative energy of four magnetic states with the variation of correlation interaction U. **f**, The electronic band structure of $NaFeO_2$(33) along non-high-symmetry directions without SOC. The red and blue lines represent the spin-up and spin-down energy bands, respectively. The electronic structure is calculated under correlation interaction U = 4 eV.

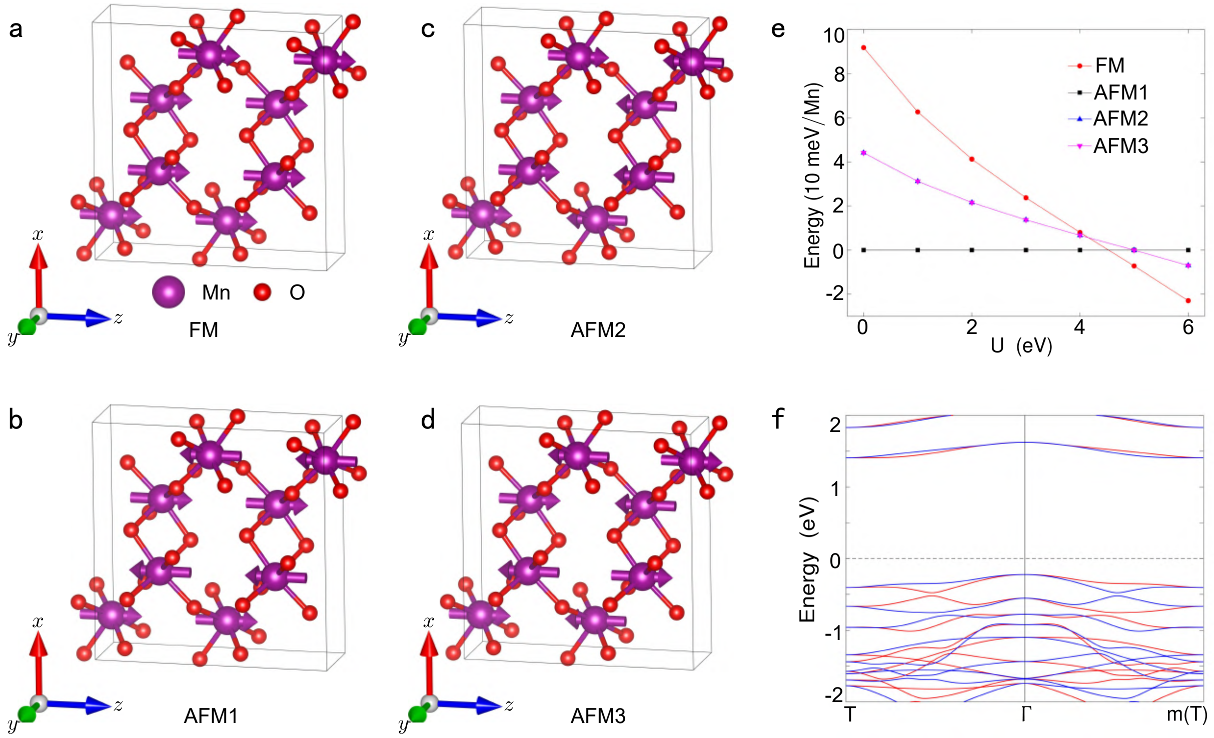

Figure S.7: Altermagnetic MnO_2(62) is confirmed by electronic structure calculations. **a**, The crystal structures of the MnO_2(62) with FM. **b**, The crystal structures of the MnO_2(62) with AFM1. **c**, The crystal structures of the MnO_2(62) with AFM2. **d**, The crystal structures of the MnO_2(62) with AFM3. The arrows represent magnetic moments of Mn. **e**, The relative energy of four magnetic states with the variation of correlation interaction U. **f**, The electronic band structure along non-high-symmetry directions without SOC. The red and blue lines represent the spin-up and spin-down energy bands, respectively. The electronic structure is calculated under correlation interaction $U = 4$ eV.

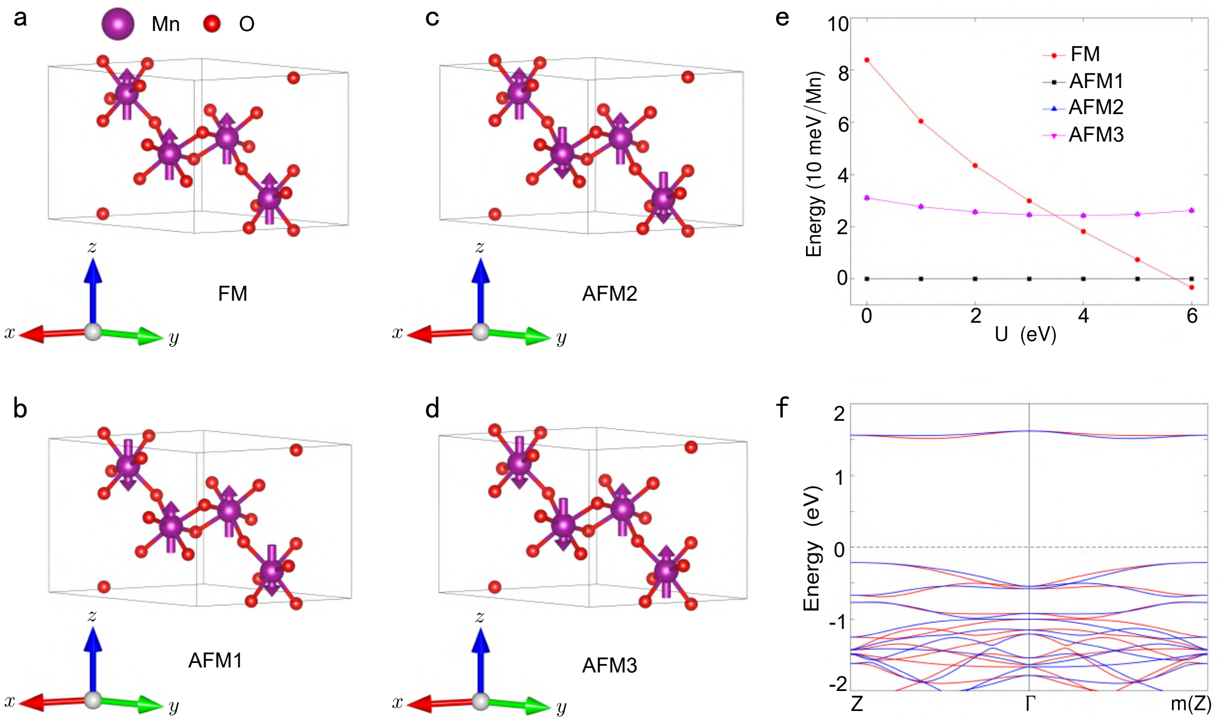

Figure S.8: Altermagnetic MnO_2(87) is confirmed by electronic structure calculations. **a**, The crystal structures of the MnO_2(87) with FM. **b**, The crystal structures of the MnO_2(87) with AFM1. **c**, The crystal structures of the MnO_2(87) with AFM2. **d**, The crystal structures of the MnO_2(87) with AFM3. The arrows represent magnetic moments of Mn. **e**, The relative energy of four magnetic states with the variation of correlation interaction U. **f**, The electronic band structure along non-high-symmetry directions without SOC. The red and blue lines represent the spin-up and spin-down energy bands, respectively. The electronic structure is calculated under correlation interaction U = 4 eV.

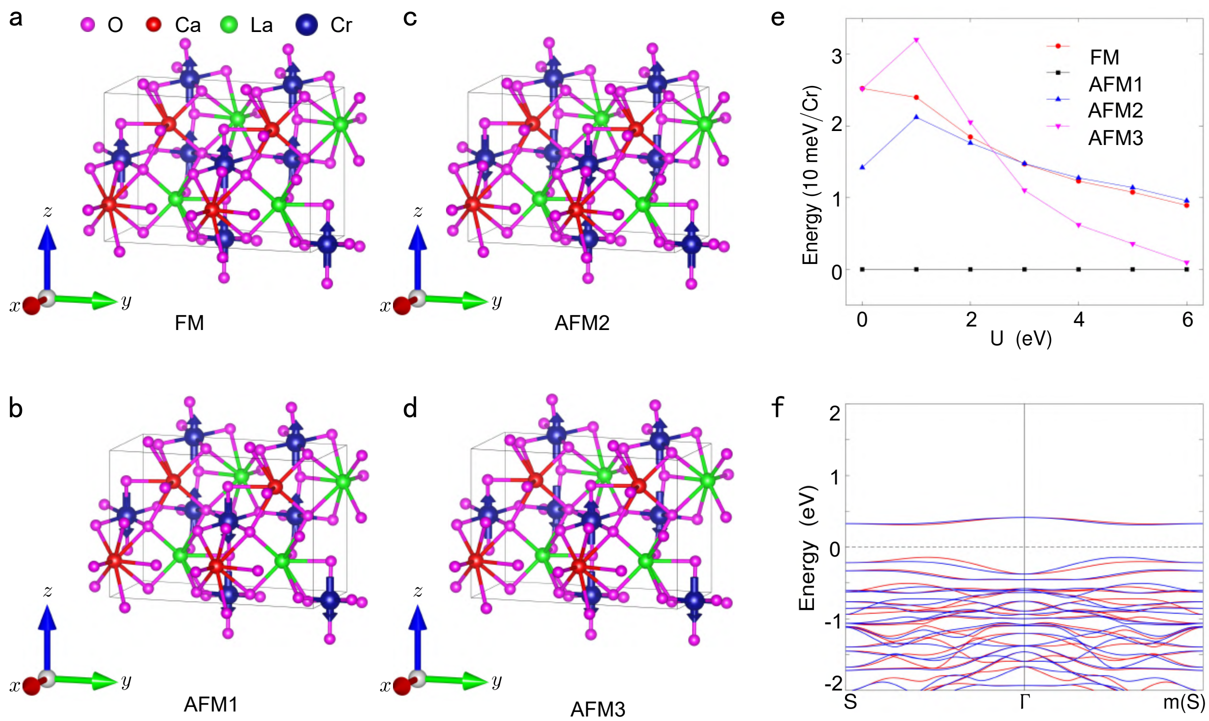

Figure S.9: Altermagnetic $CaLaCr_2O_6$ is confirmed by electronic structure calculations. **a**, The crystal structures of the $CaLaCr_2O_6$ with FM. **b**, The crystal structures of the $CaLaCr_2O_6$ with AFM1. **c**, The crystal structures of the $CaLaCr_2O_6$ with AFM2. **d**, The crystal structures of the $CaLaCr_2O_6$ with AFM3. The arrows represent magnetic moments of Cr. **e**, The relative energy of four magnetic states with the variation of correlation interaction U. **f**, The electronic band structure along non-high-symmetry directions without SOC. The red and blue lines represent the spin-up and spin-down energy bands, respectively. The electronic structure is calculated under correlation interaction U = 4 eV.

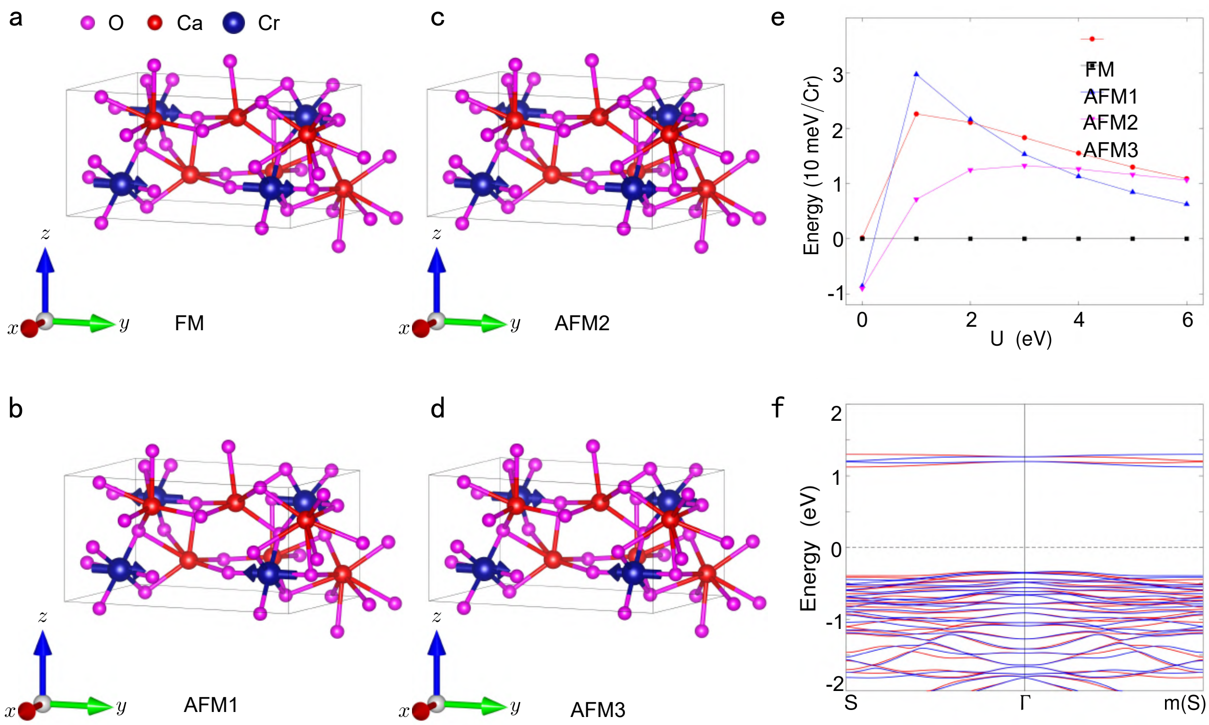

Figure S.10: Altermagnetic $Ca_3Cr_2O_7$ is confirmed by electronic structure calculations. **a**, The crystal structures of the $Ca_3Cr_2O_7$ with FM. **b**, The crystal structures of the $Ca_3Cr_2O_7$ with AFM1. **c**, The crystal structures of the $Ca_3Cr_2O_7$ with AFM2. **d**, The crystal structures of the $Ca_3Cr_2O_7$ with AFM3. The arrows represent magnetic moments of Cr. **e**, The relative energy of four magnetic states with the variation of correlation interaction U. **f**, The electronic band structure along non-high-symmetry directions without SOC. The red and blue lines represent the spin-up and spin-down energy bands, respectively. The electronic structure is calculated under correlation interaction U = 4 eV.

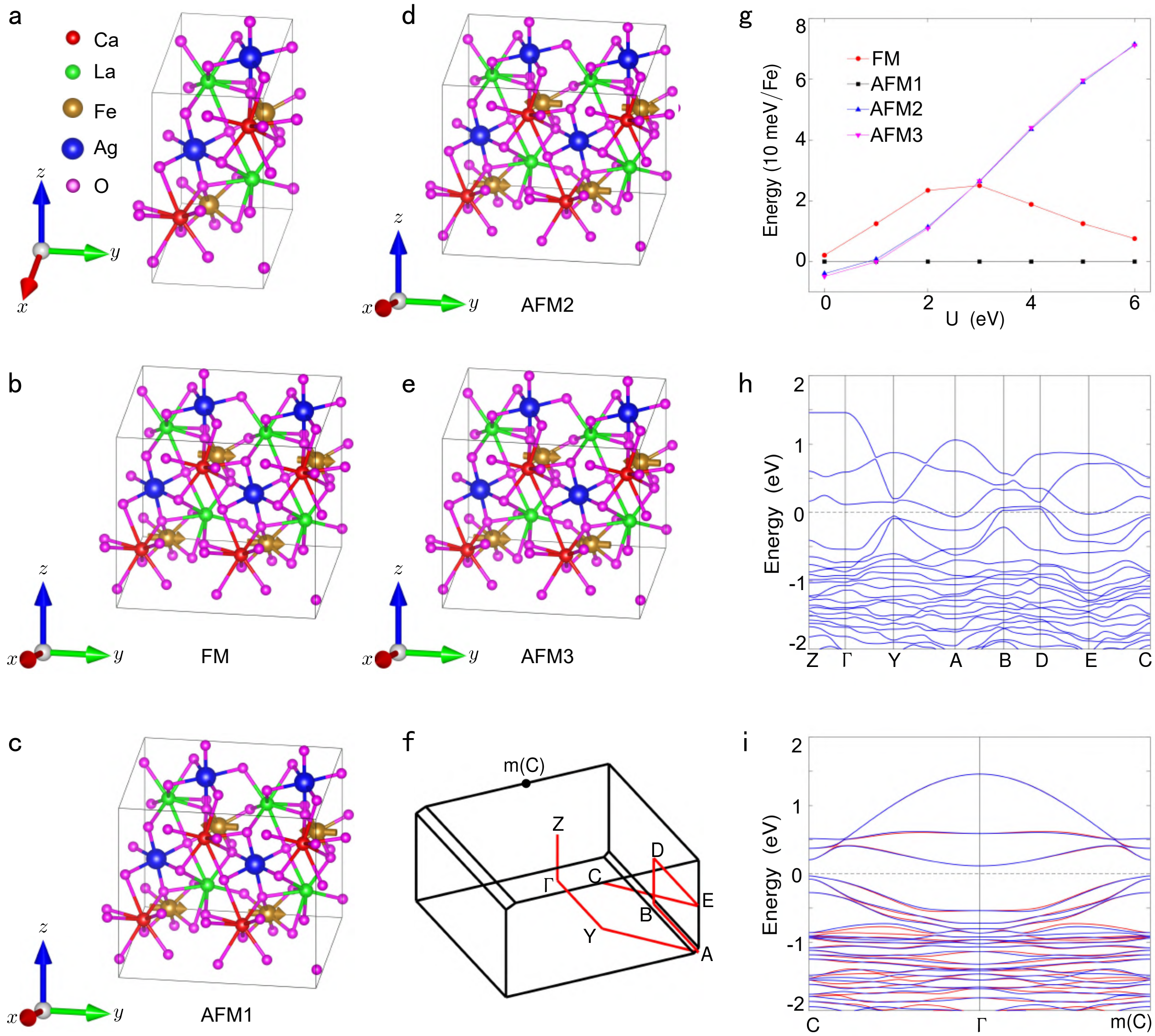

Figure S.11: The crystal and electronic structure of the altermagnetic $CaLaFeAgO_6$. **a**, The crystal primitive cell of altermagnetic $CaLaFeAgO_6$. **b**, The crystal structures of the $CaLaFeAgO_6$ with FM. **c**, The crystal structures of the $CaLaFeAgO_6$ with AFM1. **d**, The crystal structures of the $CaLaFeAgO_6$ with AFM2. **e**, The crystal structures of the $CaLaFeAgO_6$ with AFM3. The arrows represent magnetic moments of Fe. **f**, The Brillouin zone (BZ) with high-symmetry points and lines of altermagnetic $CaLaFeAgO_6$. **g**, The relative energy of four magnetic states with the variation of correlation interaction U. **h**, The electronic band structure along high-symmetry directions without SOC. **i**, The electronic band structure along non-high-symmetry directions without SOC. The red and blue lines represent the spin-up and spin-down energy bands, respectively. The electronic structure is calculated under correlation interaction $U = 4$ eV.

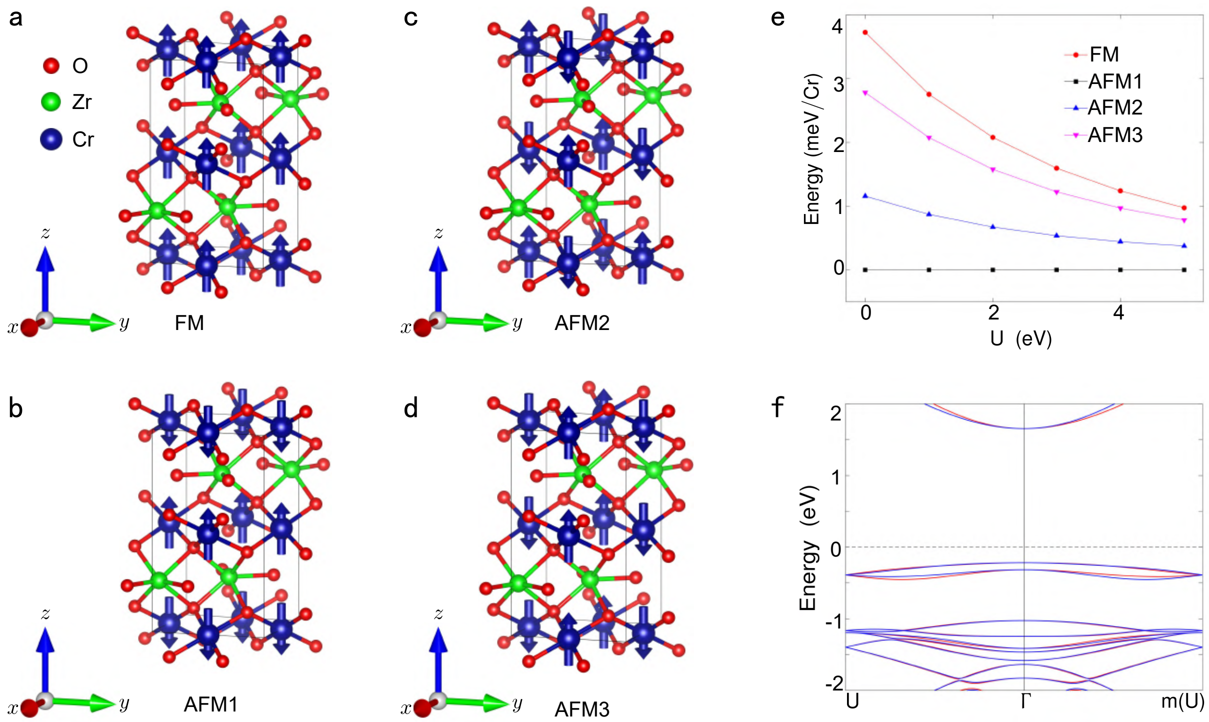

Figure S.12: Altermagnetic $ZrCrO_3$ is confirmed by electronic structure calculations. **a**, The crystal structures of the $ZrCrO_3$ with FM. **b**, The crystal structures of the $ZrCrO_3$ with AFM1. **c**, The crystal structures of the $ZrCrO_3$ with AFM2. **d**, The crystal structures of the $ZrCrO_3$ with AFM3. The arrows represent magnetic moments of Cr. **e**, The relative energy of four magnetic states with the variation of correlation interaction U. **f**, The electronic band structure along non-high-symmetry directions without SOC. The red and blue lines represent the spin-up and spin-down energy bands, respectively. The electronic structure is calculated under correlation interaction U = 4 eV.

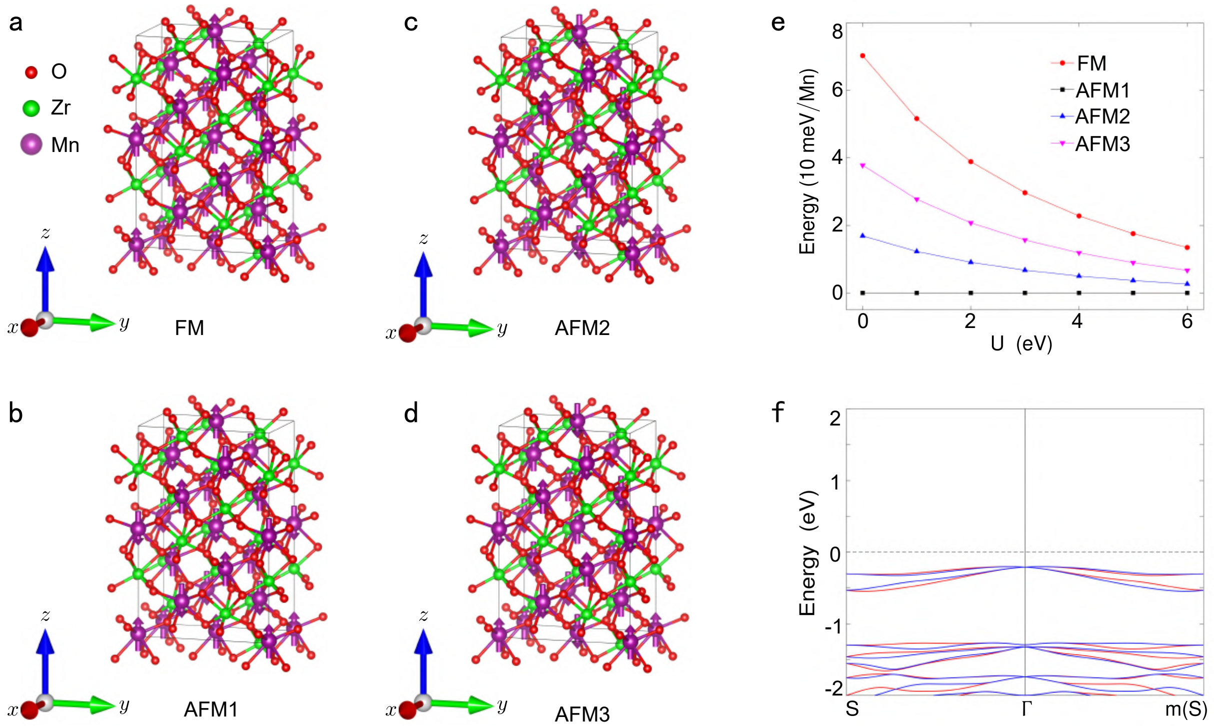

Figure S.13: Altermagnetic $ZrMnO_3$ is confirmed by electronic structure calculations. **a**, The crystal structures of the $ZrMnO_3$ with FM. **b**, The crystal structures of the $ZrMnO_3$ with AFM1. **c**, The crystal structures of the $ZrMnO_3$ with AFM2. **d**, The crystal structures of the $ZrMnO_3$ with AFM3. The arrows represent magnetic moments of Mn. **e**, The relative energy of four magnetic states with the variation of correlation interaction U. **f**, The electronic band structure along non-high-symmetry directions without SOC. The red and blue lines represent the spin-up and spin-down energy bands, respectively. The electronic structure is calculated under correlation interaction U = 4 eV.

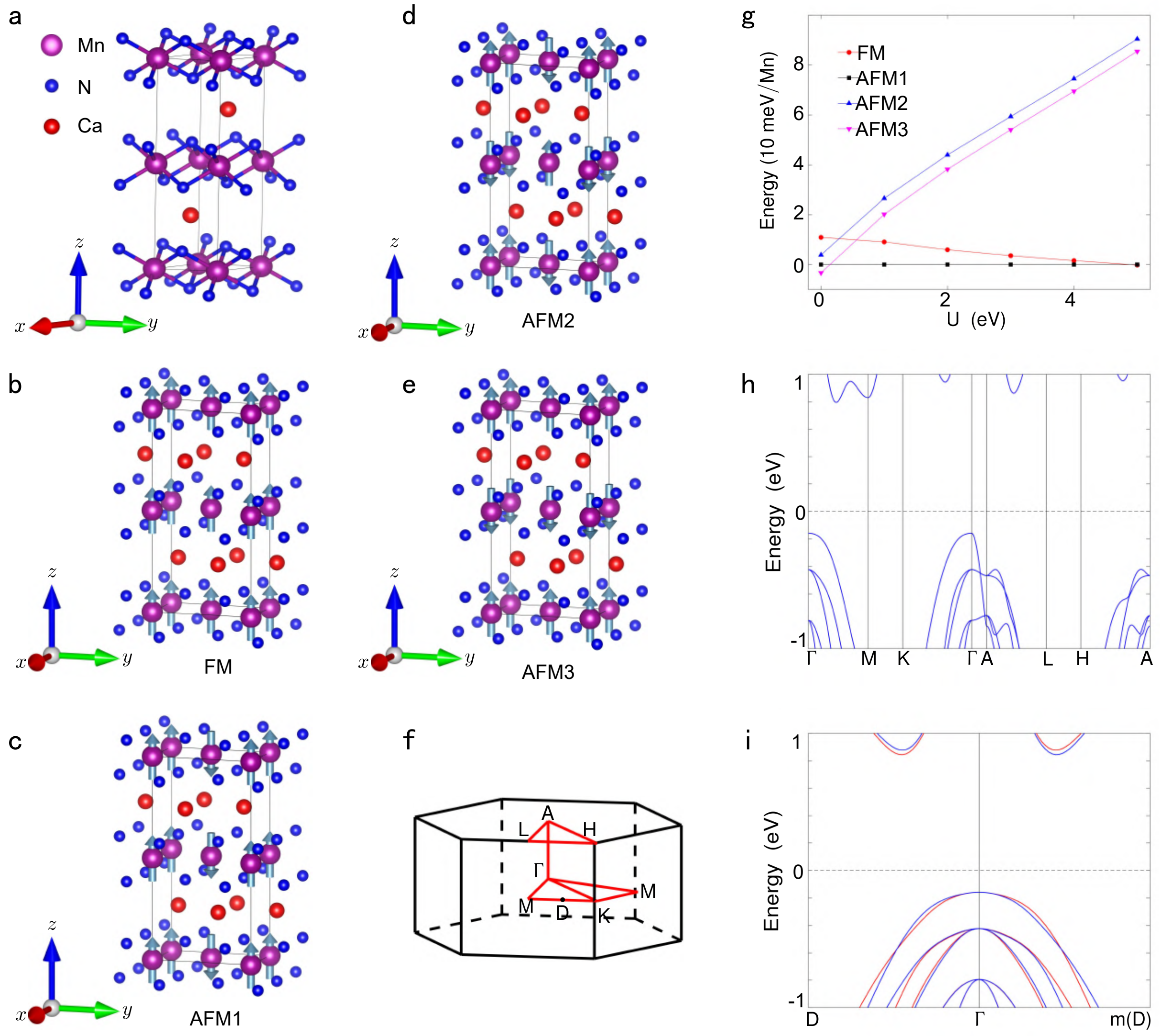

Figure S.14: The crystal and electronic structure of the altermagnetic $CaMnN_2$. **a**, The crystal primitive cell of altermagnetic $CaMnN_2$. **b**, The crystal structures of the $CaMnN_2$ with FM. **c**, The crystal structures of the $CaMnN_2$ with AFM1. **d**, The crystal structures of the $CaMnN_2$ with AFM2. **e**, The crystal structures of the $CaMnN_2$ with AFM3. The arrows represent magnetic moments of Mn. **f**, The Brillouin zone (BZ) with high-symmetry points and lines of altermagnetic $CaMnN_2$. **g**, The relative energy of four magnetic states with the variation of correlation interaction U. **h**, The electronic band structure along high-symmetry directions without SOC. **i**, The electronic band structure along non-high-symmetry directions without SOC. The red and blue lines represent the spin-up and spin-down energy bands, respectively. The electronic structure is calculated under correlation interaction U = 4 eV.

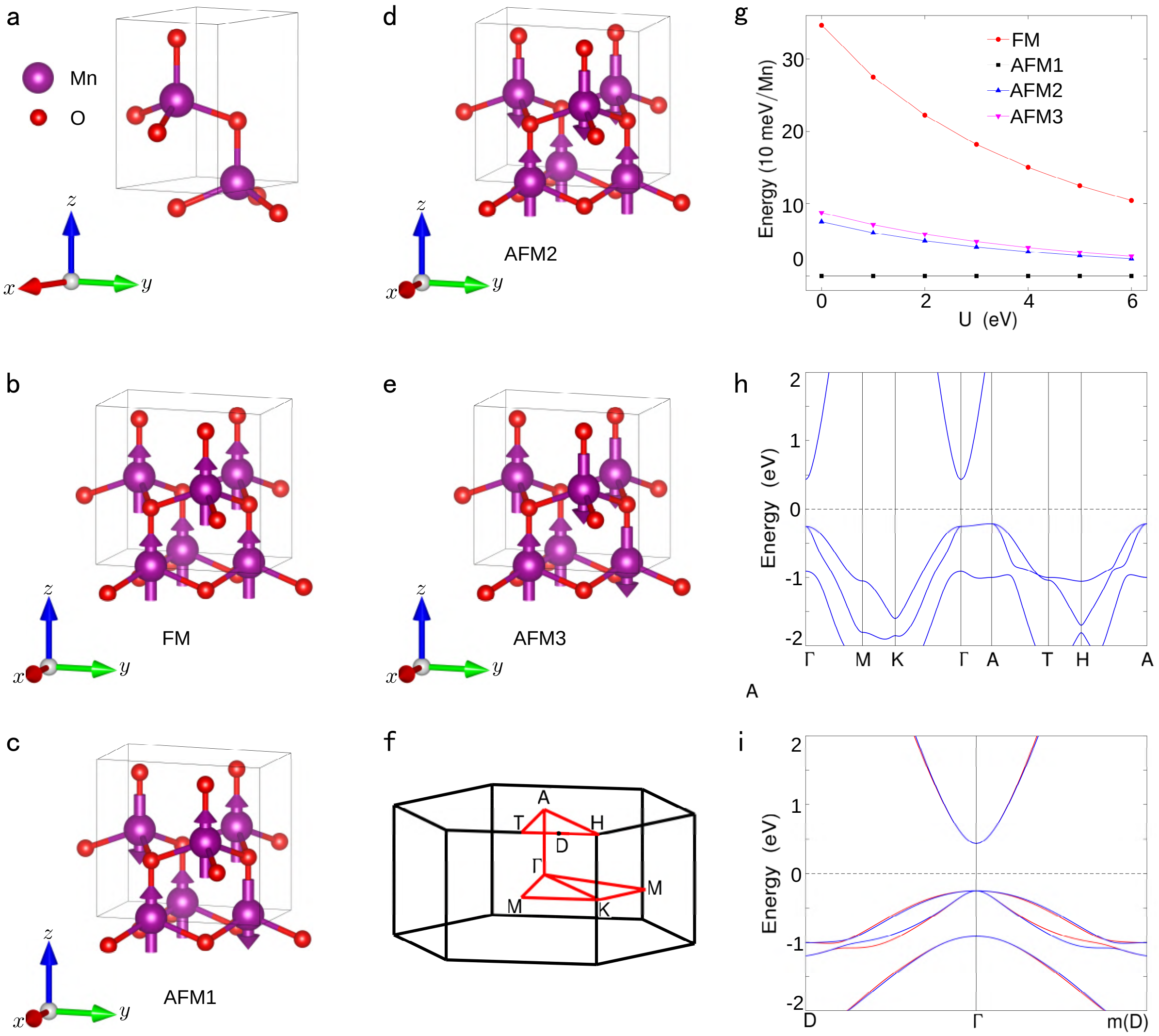

Figure S.15: The crystal and electronic structure of the altermagnet MnO. **a**, The crystal primitive cell of altermagnetic MnO. **b**–**e**, The four significant magnetic structures MnO. The arrows represent magnetic moments of Mn. **f**, The Brillouin zone (BZ) with high-symmetry points and lines of altermagnetic MnO. **g**, The relative energy of four significant magnetic states with the variation of correlation interaction U. **h** and **i**, The electronic band structure of MnO along high-symmetry directions and non-high-symmetry directions without spin-orbit coupling (SOC), respectively. The red and blue lines represent the spin-up and spin-down energy bands, respectively. The electronic structure is calculated under correlation interaction U = 4 eV.

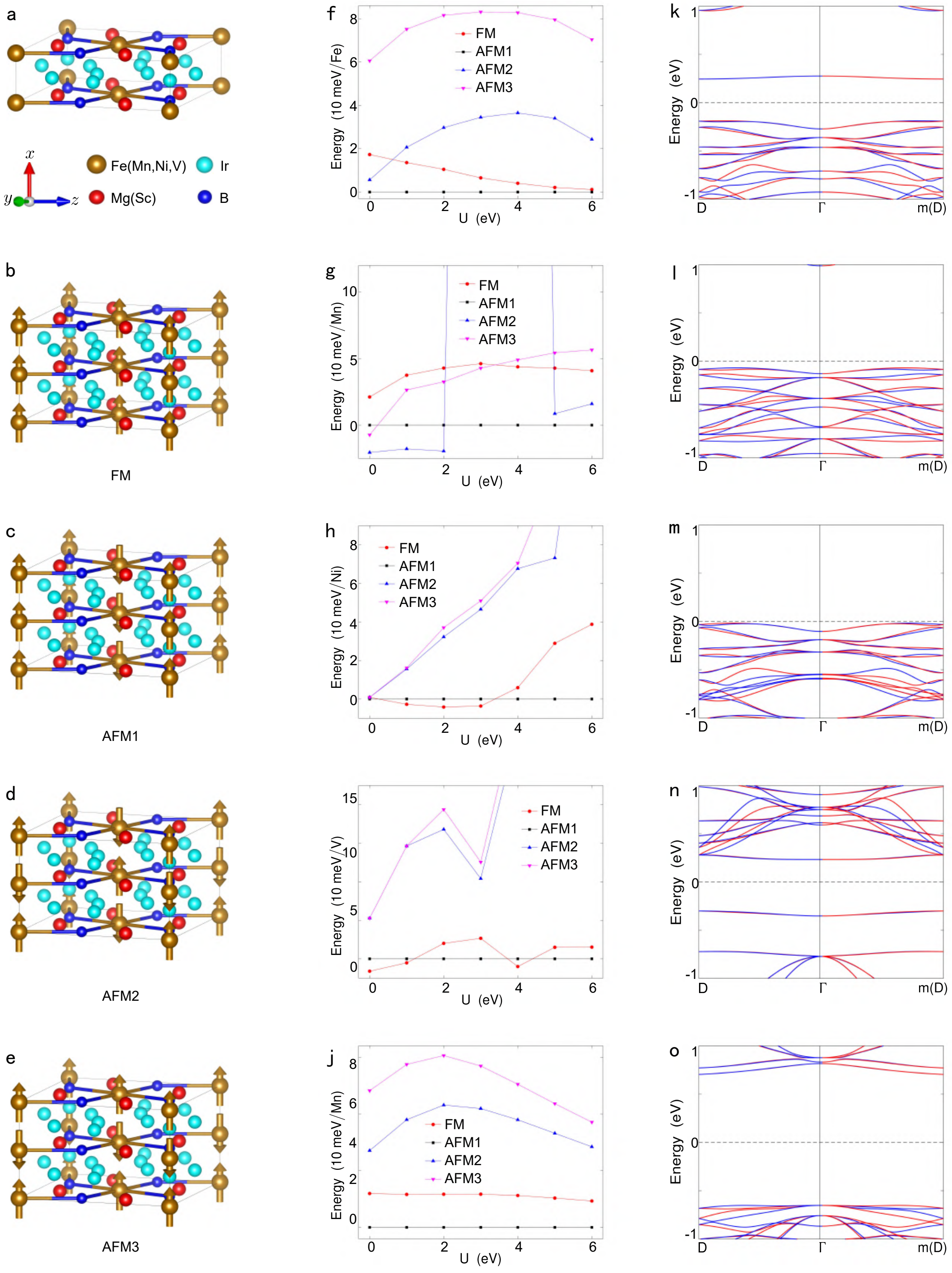

Figure S.16: Altermagnetic materials are confirmed by electronic structure calculations. a, The crystal primitive cell. **b**–**e**, The crystal structures of the $Ir_5B_2Y_2X$ (Y=Mg,Sc,X=Fe,Mn,Ni,V) with FM, AFM1, AFM2 and AFM3 magnetic structures, respectively. **f**–**j**, Relative energy of four significant magnetic states with the variation of correlation interaction U of $Ir_5B_2Mg_2Fe$, $Ir_5B_2Mg_2Mn$, $Ir_5B_2Mg_2Ni$, $Ir_5B_2Sc_2V$ and $Ir_5B_2Sc_2Mn$. **k**–**o**, The electronic band structure of altermagnetic $Ir_5B_2Y_2X$ along non-high-symmetry directions without SOC. The red and blue lines represent spin-up and spin-down energy bands, respectively.

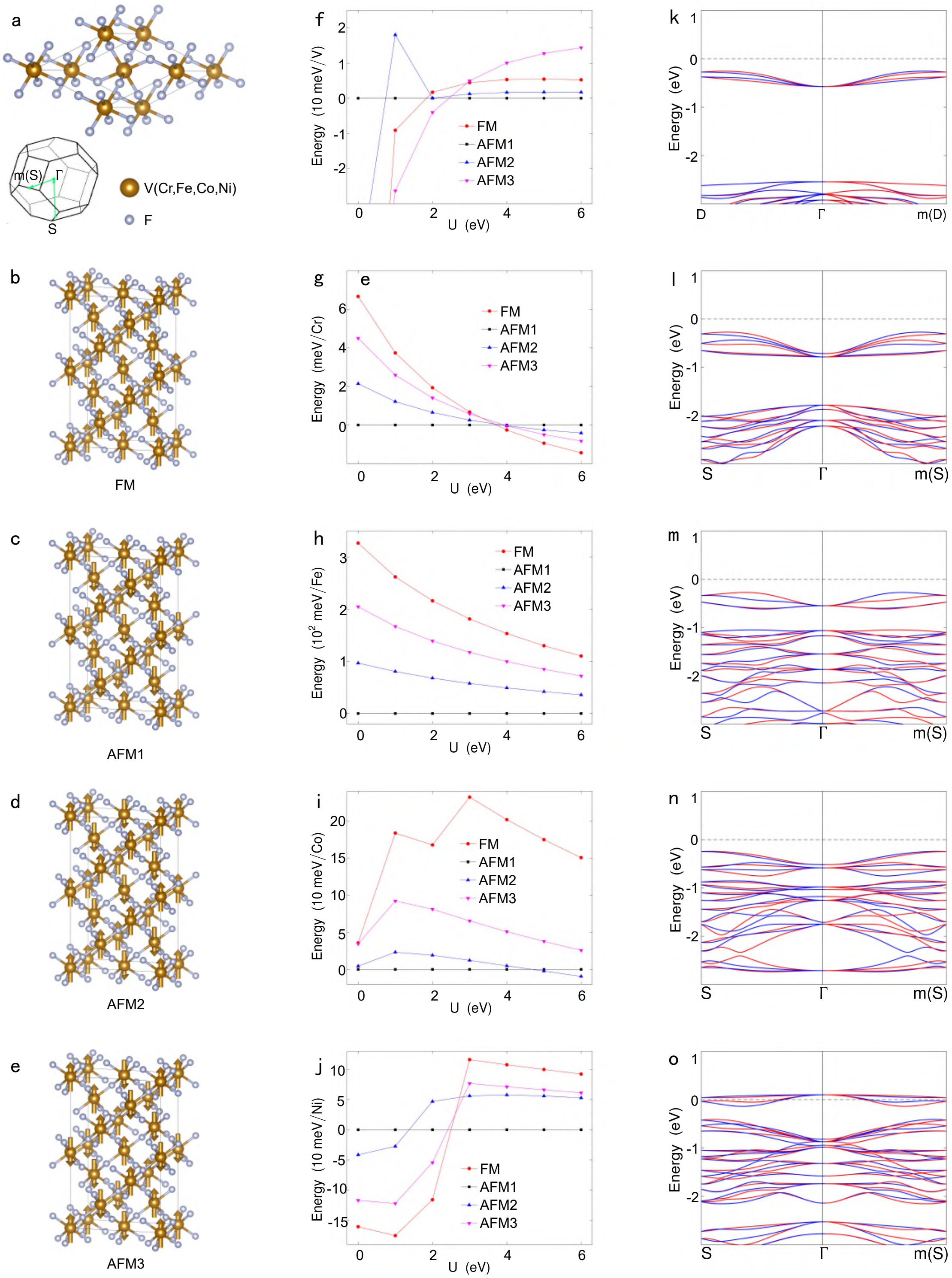

Figure S.17: Altermagnetic materials are confirmed by electronic structure calculations. a, The crystal primitive cell and Brillouin Zone (BZ). **b–e**, The crystal structures of XF_3 (X=V,Cr,Ni) with FM, AFM1, AFM2 and AFM3 magnetic structures, respectively. **f–j**, Relative energy of four significant magnetic states with the variation of correlation interaction U of VF_3, CrF_3, and NiF_3. **k–o**, The electronic band structure of altermagnetic XF_3 along non-high-symmetry directions without SOC. The red and blue lines represent spin-up and spin-down energy bands, respectively. The electronic structure is calculated under correlation interaction U = 4 eV.

Table S.2: The band gap of the 34 altermagentic insulator materials under the LDA+U framework.

Number	Materials	Gap/eV
1	$FeHO_2$(31)	1.94
2	$NaFeO_2$ (33)	3.18
3	$NaFeO_2$ (92)	3.11
4	MnO_2 (62)	1.60
5	MnO_2 (87)	1.73
6	$Ca_3Cr_2O_7$	1.34
7	$ZrCrO_3$	1.86
8	$ZrMnO_3$	3.47
9	VF_3	2.87
10	CrF_3	4.13
11	MnO	0.65
12	$CaMnN_2$	0.95
13	VF_4	3.69
14	Ca_2CoTeO_6	2.04
15	NiF_2	4.70
16	$Ba_2FeGe_2O_7$	1.85
17	$Ba_2CoSi_2O_7$	3.15
18	$Sr_2CoGe_2O_7$	1.93
19	$FeHO_2$(19)	2.26
20	$CaMnO_3$	1.21
21	$CaVO_3$	1.11
22	$LaFeO_3$	2.45
23	$LaVO_3$	2.22
24	$MnSeO_4$	2.74
25	$NaPr_2OsO_6$	0.45
26	$NaPr_2RuO_6$	0.76
27	$NdRhO_3$	1.47
28	$PrRuO_3$	0.18
29	$ScVO_3$	2.30
30	$SmRhO_3$	1.18
31	$CaLaCrMoO_6$	0.83
32	$La2MnRhO_6$	0.44
33	$LiFeF_4$	3.60
34	$LiFe_2F_6$	1.55

C Addition discussion for proposed AI search engine

Intrinsic errors and computational costs associated with the proposed algorithm. About the intrinsic errors and computational costs associated with the algorithm, we provide a detailed breakdown of each computational step:

(1) **Dataset Collection** – *collecting the pre-training dataset, fine-tuning dataset, and candidate materials through high-throughput screening*: This step requires relatively less efforts since data can be fetched using the API provided by the Material Project.

(2) **Model Pre-training** – *establishing and pre-training the GNN model using the pre-training dataset*: This step allows to learn the intrinsic features of the crystal structure of magnetic materials. The computational cost of this step is mainly related to the volume of pre-training data, the size of the GNN model, and the number of training iterations. In our experiments, the training was conducted using four Nvidia A100 GPUs, taking approximately 35 hours to converge.

(3) **Classifier Fine-tuning** – *fine-tuning the classifier model using the fine-tuning dataset*: This step allows the model to predict altermagnetism probability of a given material candidate. Possible intrinsic errors here may arise from differences between the estimated distribution of the positive samples and the true distribution, which can lead to biased prediction by the model. This can be mitigated by iteratively adding more positive samples (e.g., once a new altermagnetic material is discovered, it is added to the list of positive samples to refine the classifier model). Since there are quite a limited number of positive samples, the training effort in this step typically takes about 2–3 hours on one Nvidia A100 GPU.

(4) **Material Verification** – *conducting DFT electronic structure calculations on the predicted candidate materials to verify their altermagnetic properties*: This step involves determining the magnetic ground state and band structure, whose computational cost depends on factors such as the number of atoms in the primitive cell. Typically, the calculation of each material for verification takes about 12–48 hours. Note that the calculations were conducted on a server equipped with 64-core Intel Xeon Gold 6438 processors with parallel computing.

AI component *v.s.* simply searching based on physics. The “brute-force” search based on physics involves guessing potential crystal materials by experts followed by DFT calculations for verification, which are not very quantifiable in terms of accuracy and efficiency. This conventional strategy is undesirable given tens of thousands of candidate materials. In the contrary, AI models have two advantages: (1) they rely less on experts’ experience, which leverage known positive samples for training and prediction; (2) through 3 active learning, the predicted positive samples can be further added to the training data to iteratively improve the accuracy of the model’s prediction.

The convergence of the proposed AI search engine. In our experiments, after the four rounds of iteration, the candidate materials predicted by the model predominantly consist of materials with a huge number of atoms in the unit cell[1]). In other words, we can no longer determine whether these materials exhibit altermagnetic properties (although they may possess them) through finite DFT calculations. Therefore, after four iterations, we concluded that the model had converged and could not predict additional altermagnetic materials.

[1]We consider crystalline materials with unit cells containing 40 or more atoms as materials with a huge number of atoms within the unit cell.

The few-shot learning ability of proposed AI search engine. The AI search engine can obtain predictability by fine-tuning procedures. The model performance was positive relative to the number of positive samples. In our experiment, we predicted 25 altermagnetic materials with only 14 positive samples. This result shows the few-shot learning ability of the AI search engine. Moreover, we added the rest 134 positive samples into the fine-tuned dataset, and then the model performance was significantly improved. Note that the model's performance is not directly related to spin patterns, and the model cannot directly distinguish between AFM1, AFM2, and AFM3 spin patterns.

Accuracy of the classifier model. Based on our current results, the accuracy of finding altermagnetic materials using the AI search engine is around 31% (e.g., 50 new altermagnetic materials were found, out of 161 candidate materials predicted by AI which were calculated by DFT). The accuracy of the classifier model might be improved by adding more positive samples. In contrast, when we evaluated 10 materials recommended by human experts, we could not discover any new altermagnetic properties. Such a process depends on the expertise of experts and may yield easily a biased estimation.

D Addition information for DFT calculations

We use the first principle of electronic calculations to verify the predicted materials by the AI search engine whether it is altermagnetic. Table S.3 lists the hyper-parameters for the density wave functions (DFT) in our main experiments.

Table S.3: The hyper-parameter for DFT calculations.

Number	Materials	Space group	ENCUT/eV	$\boldsymbol{k}$-grid	U-value/eV	Calculation μ_B
1	Nb_2FeB_2	$P4-mbm$ (127)	600	$8\times8\times15$	Fe:5	Fe:2.93
2	Ta_2FeB_2	$P4-mbm$ (127)	600	$8\times8\times15$	Fe:5	Fe:2.80
3	NdB_2C_2	$P4-mbm$ (127)	600	$10\times10\times16$	Nd:7	Nd:3.06
4	$Mg_2FeIr_5B_2$	$P4-mbm$ (127)	600	$6\times6\times18$	Fe:4	Fe:3.52
5	$Mg_2MnIr_5B_2$	$P4-mbm$ (127)	600	$6\times6\times18$	Mn:4	Mn:3.98
6	$Mg_2NiIr_5B_2$	$P4-mbm$ (127)	600	$6\times6\times18$	Ni:6.6	Ni:1.33
7	$Sc_2VIr_5B_2$	$P4-mbm$ (127)	600	$6\times6\times18$	V:3	V:1.21
8	$Sc_2MnIr_5B_2$	$P4-mbm$ (127)	600	$6\times6\times18$	Mn:4	Mn:4.14
9	$CaLaFeAgO_6$	Pc (7)	600	$12\times8\times6$	Fe:4	Fe:3.85
10	$CaLaCr_2O_6$	$Pmn2_1$ (31)	600	$10\times10\times8$	Cr:4	Cr:2.58
11	NiF_3	$R\bar{3}C$ (167)	600	$16\times16\times16$	Ni:6.7	Ni:1.53
12	GdB_2C_2	$P4/mbm$ (127)	600	$8\times8\times14$	Gd:7	Gd:7.15
13	HoB_2C_2	$P4/mbm$ (127)	600	$8\times8\times14$	Ho:7	Ho:4.09
14	$LuCrO_3$	$Pnma$ (62)	600	$10\times10\times8$	Lu:7;Cr:4	Lu:0.00;Cr:0.97
15	$TaCoB_2$	$Pnma$ (62)	600	$8\times16\times6$	Co:3	Co:1.13
16	$NdRuO_3$	$Pnma$ (62)	600	$8\times8\times10$	Nd:7;Ru:2	Nd:3.00;Ru:0.73
17	$FeHO_2$	$Pmn2_1$ (31)	600	$18\times12\times10$	Fe:4	Fe:4.16
18	$NaFeO_2$	$Pna2_1$ (33)	600	$10\times8\times10$	Fe:4	Fe:4.01
19	$NaFeO_2$	$P4_12_12$ (92)	600	$10\times10\times8$	Fe:4	Fe:3.98
20	MnO_2	$Pnma$ (62)	600	$6\times18\times12$	Mn:4	Mn:3.04
21	MnO_2	$I4/m$ (87)	600	$8\times8\times4$	Mn:4	Mn:3.03
22	$Ca_3Cr_2O_7$	$Cmc2_1$ (36)	600	$10\times10\times10$	Cr:4	Cr:2.06
23	$ZrCrO_3$	$Pnma$ (62)	600	$10\times10\times6$	Cr:4	Cr:3.73
24	$ZrMnO_3$	$R3c$ (161)	600	$12\times12\times12$	Mn:4	Mn:4.59
25	VF_3	$R\bar{3}C$ (167)	600	$16\times16\times16$	V:3	V:1.87
26	CrF_3	$R\bar{3}C$ (167)	600	$16\times16\times16$	Cr:3	Cr:2.86
27	MnO	$P6_3mc$ (186)	600	$18\times18\times10$	Mn:4	Mn:4.52
28	$CaMnN_2$	$P6_3/mmc$ (194)	600	$18\times18\times6$	Mn:4	Mn:3.39
29	$Ba_2FeGe_2O_7$	$P\bar{4}2_1m$ (113)	600	$6\times6\times8$	Fe:5	Fe:3.69
30	$Ba_2CoSi_2O_7$	$P\bar{4}2_1m$ (113)	600	$6\times6\times8$	Co:3	Co:2.69
31	$Sr_2CoGe_2O_7$	$P\bar{4}2_1m$ (113)	600	$6\times6\times8$	Co:3	Co:2.67
32	VF_4	$P2_1/c$ (14)	600	$12\times6\times12$	V:4	V:1.00
33	Ca_2CoTeO_6	$P2_1/c$ (14)	600	$8\times8\times6$	Co:3	Co:2.69
34	NiF_2	$Pnnm$ (58)	600	$10\times10\times16$	Ni:5	Ni:1.81
35	$LiFe_2F_6$	$P4_2nm$ (102)	600	$10\times10\times6$	Fe:5	Fe:4.088
36	$FeHO_2$	$P2_12_12_1$ (19)	600	$16\times12\times6$	Fe:5	Fe:4.27
37	$CaMnO_3$	$Pnma$ (62)	600	$10\times8\times10$	Mn:4	Mn:2.94
38	$CaVO_3$	$Pnma$ (62)	600	$10\times8\times10$	V:4	V:0.95
39	$LaFeO_3$	$Pnma$ (62)	600	$10\times10\times6$	Fe:5	Fe:4.18
40	$LaVO_3$	$Pnma$ (62)	600	$10\times6\times10$	V:4	V:1.85
41	$MnSeO_4$	$Pnma$ (62)	600	$10\times6\times8$	Mn:4	Mn:4.63
42	$NaPr_2OsO_6$	$P2_1/c$ (14)	600	$10\times8\times6$	Pr:7;Os:2	Pr:2.00;Os:2.16
43	$NaPr_2RuO_6$	$P2_1/c$ (14)	600	$10\times8\times6$	Pr:7;Ru:2	Pr:2.00;Ru:0.67
44	$NdRhO_3$	$Pnma$ (62)	600	$10\times6\times10$	Nd:7;Rh:2	Nd:3.00;Rh:0.00
45	$PrRuO_3$	$Pnma$ (62)	600	$10\times6\times10$	Pr:7;Ru:2	Pr:2.00;Ru:0.71
46	$ScVO_3$	$Pnma$ (62)	600	$10\times8\times10$	V:4	V:1.83
47	$SmRhO_3$	$Pnma$ (62)	600	$10\times6\times10$	Sm:7;Rh:2	Sm:5.08;Rh:0.00
48	$CaLaCrMoO_6$	Pc (7)	600	$10\times8\times6$	Cr:5;Mo:2	Cr:2.93;Mo:1.67
49	La_2MnRhO_6	$P2_1/c$ (14)	600	$10\times8\times6$	Mn:5;Rh:2	Mn:3.99;Rh:0.10
50	$LiFeF_4$	$P2_1/c$ (14)	600	$10\times10\times10$	Fe:5	Fe:4.4
